# Unraveling the effect of choline-based choline based ionic liquids on the physicochemical properties and taste behavior of *D(* +*)*-glucose in aqueous solutions

**DOI:** 10.1186/s13065-025-01407-3

**Published:** 2025-02-22

**Authors:** Sara Dorosti, Hemayat Shekaari, Mohammad Bagheri, Fariba Ghaffari, Masumeh Mokhtarpour

**Affiliations:** Department of Physical Chemistry, Faculty of Chemistry, University of Tabriz, Tabriz, Iran

**Keywords:** Choline based-ionic liquids, *D(* +*)*-glucose, Thermodynamic properties, Taste behavior, DFT-COSMO calculations

## Abstract

To elucidate the mechanism by which choline-based ionic liquids potentially can enhance the sugar conversion to bioethanol, this work was conducted to study the thermodynamic behavior of *D(* +*)*-glucose in aqueous solutions of choline-based ionic liquids, choline salicylate [Ch][Sal], choline formate [Ch][For], and choline acetate [Ch][Ace]. This study involved measuring density, speed of sound, viscosity, and electrical conductivity at various concentrations and temperatures. Analysis of the calculated parameters, including apparent molar volume*, V*_φ_, apparent molar isentropic compressibility (*κ*_φ_), viscosity *B*-coefficient, and molar conductivity (*Λ*) values provide deep insights into intermolecular interactions between the components of the solutions studied. The standard partial molar volume values ($V_{\varphi }^{0}$) of *D(* +*)*-glucose, show stronger interactions between *D(* +*)*-glucose and the [Ch][Sal]. The computed transfer volume values ($\Delta_{tr} V_{\varphi }^{0}$), with the help of co-sphere overlap model confirm intensified hydrophilic-hydrophilic interactions in [Ch][Sal] [(1.99 to 2.08) cm^3^·mol^−1^] solutions. Hepler's constants suggest that *D(* +*)*-glucose acts as a structure-maker in the presence of choline-based ILs, especially in [Ch][Sal] solutions. Also, the DFT-COSMO calculations result in [Ch][Sal] the most favorable interactions among the other choline based ILs. Apparent specific volume (*ASV*), and apparent specific isentropic compressibility, (*ASIC*), values revealed that *D(* +*)*-glucose exhibits the taste behavior with [Ch][Sal]. The hydration number of *D(* +*)*-glucose diminishes as the temperature rises due to weakened hydrogen bonds between *D(* +*)*-glucose and water molecules. These findings suggest that [Ch][Sal] could be a promising candidate for accelerating sugar conversion to bioethanol.

**Graphical Abstract:**

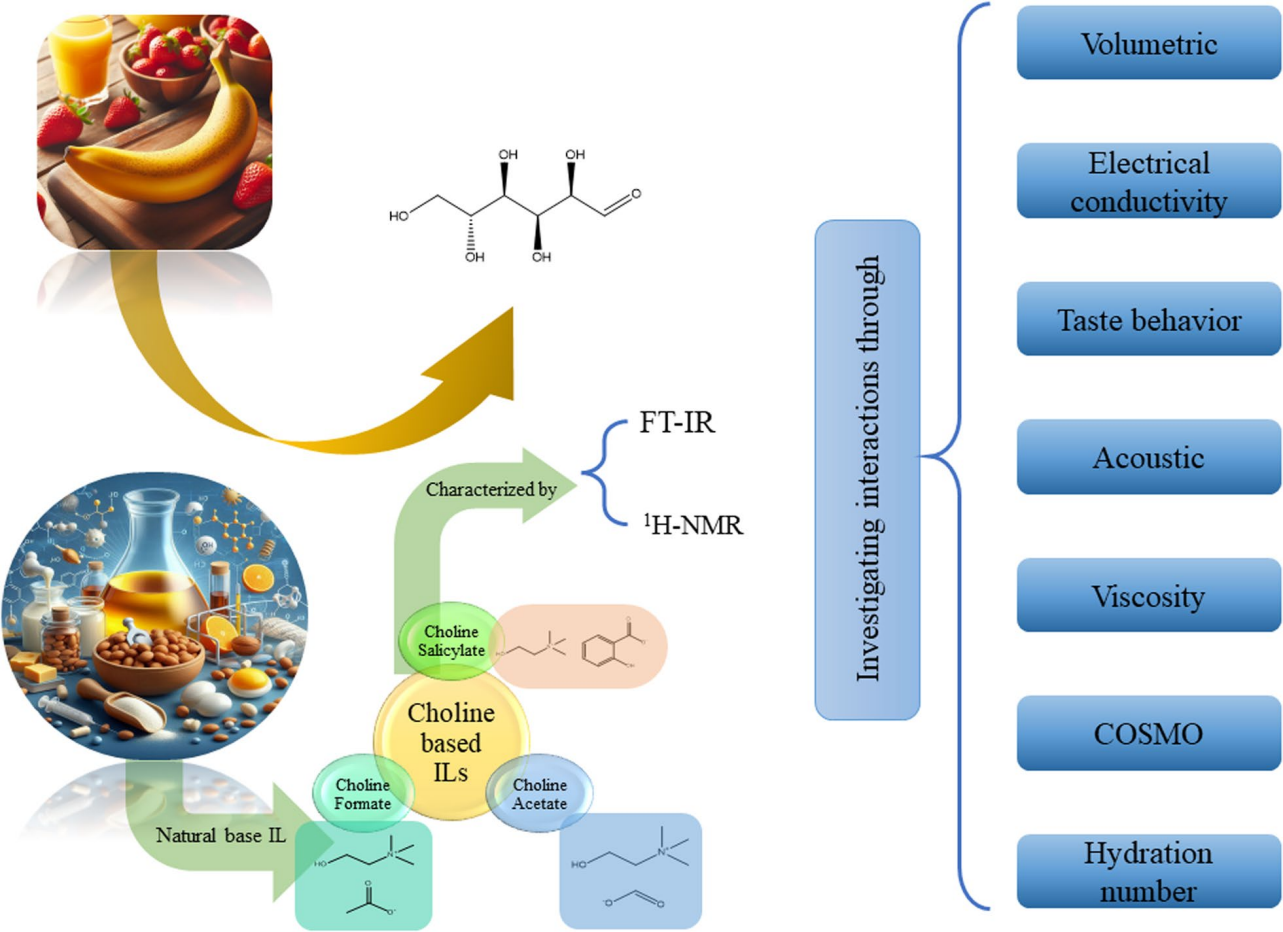

**Supplementary Information:**

The online version contains supplementary material available at 10.1186/s13065-025-01407-3.

## Introduction

Air pollution, global warming, sea level rising are amongst the most trifling problems that have set mankind's mind at unease [1, 2]. These problems are mostly due to the overconsumption of fossil fuels. There have been many solutions suggested to overcome these concerns for this purpose, it is necessary to find replaceable solutions for fossil fuels [3–5]. Bioethanol stands as promising replaceable for fossil fuels. Bioethanol, when utilized as a transportation fuel, has the potential to mitigate climate change by reducing greenhouse gas emissions. It is also biodegradable and produces fewer harmful pollutants than traditional fossil fuels, which can cause an improvement in the air quality. Moreover, bioethanol production can stimulate rural economies by creating jobs in agriculture and processing industries. Beyond environmental advantages, bioethanol enhances energy security by reducing dependence on foreign oil imports. There are various ways to produce bioethanol, one of which is the conversion of sugars into bioethanol [6, 7].

Choline based-ILs have emerged as promising catalysts for the hydrolysis step in bioethanol production, offering several advantages over traditional solvents. These unique solvents possess distinctive properties such as low vapor pressure, high ionic conductivity, and excellent solvating ability, which enable them to effectively disrupt the complex structure of lignocellulosic biomass, thereby enhancing the accessibility of cellulose to hydrolyzing enzymes. By interacting with the enzymes, Ionic liquids can also alter their conformation and improve their catalytic activity, leading to a faster conversion of glucose into bioethanol. Among the various Ionic liquids studied, choline-based-ILs have shown particular promise due to their inherent advantages [8, 9]. These compounds are derived from choline, an essential nutrient for the human body, and exhibit a high degree of biodegradability, making them more environmentally friendly than many other Ionic liquids. The presence of the choline moiety in these Ionic liquids can also confer additional benefits, such as improved compatibility with biological systems and reduced toxicity. Moreover, the tunability of choline-based ILs allows for the fine-tuning of their properties to optimize their performance in specific applications, further enhancing their potential for bioethanol production [10–12].

*D(* +*)*-glucose with a chemical formula of C_6_H_12_O_6_, is the most abundant monosaccharide and mostly can be found in plants [13]. As a simple sugar, it is the primary energy source for the human body [14]. It is derived from the carbohydrates that get consumed daily and is transported through the bloodstream to reach every cell. Inside cells, glucose undergoes a complex process called cellular respiration, producing the energy needed for various bodily functions such as muscle movement, brain activity, and organ function [15]. Maintaining stable glucose levels is crucial for optimal health, as imbalances can lead to serious conditions like hypoglycemia (low blood sugar) or hyperglycemia (high blood sugar), associated with diabetes [16, 17]. The conversion of sugars (glucose) into bioethanol usually consists of two main stages of hydrolysis and fermentation. In the hydrolysis complex carbohydrates like cellulose and hemicellulose gets break down into smaller sugars, like glucose. This step is mostly facilitated by enzymes or acids [16, 18–20]. Fermentation process on the other hand, involves utilization of microorganisms such as yeast to convert glucose into ethanol and carbon dioxide under anaerobic conditions [21, 22]. This metabolic pathway, known as glycolysis, involves a series of enzymatic reactions that gradually break down glucose into pyruvate, which is then transformed into ethanol [23]. These procedures are time-intensive, and researchers have made numerous efforts to expedite them. For example, the use of organic solvents has been studied to facilitate the breakdown of sugar bonds; however, these solvents are accompanied by inherent limitations despite yielding favorable outcomes. Consequently, choline based-ILs have emerged as a preferred option due to their comparative advantages over other solvents [11, 24–27]. Choline based-ILs have emerged as promising catalysts for the hydrolysis step in bioethanol production [28]. These unique solvents possess distinctive properties, including low vapor pressure, high ionic conductivity, and excellent solvating ability, and the most important their tunability factors [29]. By disrupting the complex structure of lignocellulosic biomass, choline based-ILs can enhance the accessibility of cellulose to hydrolyzing enzymes, thereby accelerating the glucose release process [30, 31]. Moreover, ILs can interact with the enzymes, altering their conformation and improving their catalytic activity. These combined effects significantly contribute to the faster conversion of glucose into bioethanol, making ILs a valuable tool for enhancing the efficiency of bioethanol production processes [32–34]. Choosing the right type of ILs that is the most suitable for the task is most important, as ILs must have biodegradable and eco-friendly aspect to not harm the ecosystem any more. Choline based ILs are amongst the greenest, substances that can be categorized as the third generation of ILs and count as aprotic ILs. The choline part of the ILs is an essential nutrient that plays a important part in various bodily functions [35]. It's particularly vital for brain health, as it has been involved in the production of acetylcholine, a neurotransmitter essential for memory, mood, and muscle control [36–38].

Interfacial electron density serves as a fundamental parameter in analyzing molecular surface characteristics and can be evaluated using density functional theory (DFT) computations. The conductor-like screening model (COSMO), integrated within the Dmol3 computational framework, provides a reliable and efficient approach for determining various molecular descriptors. This method enables the estimation of total surface cavity area (*A*), cavity volume (*V*), and dielectric solvation energy, along with electronic properties such as the highest occupied molecular orbital (HOMO) and the lowest unoccupied molecular orbital (LUMO). Moreover, the COSMO model generates a *σ*-profile, which reflects the dielectric behavior of the molecular structure [39, 40]. To assess the efficiency of choline-based ILs in sugar conversion to bioethanol, it is imperative to understand the intermolecular interactions between the choline-based ILs and the sugars [41–44].

For this purpose, in this study, the series of experiments were performed to evaluate the effectiveness of choline-based ILs in the presence of *D(* +*)*-glucose. Specifically, the volumetric, acoustic, viscometric and conductometric properties of *D(* +*)*-glucose in aqueous solutions containing choline-based ILs were investigated. The density (*ρ*), speed of sound (*u*), viscosity (*η*), and electrical conductivity data were measured. The three choline-based ILs are choline salicylate ([Ch][Sal]), choline formate ([Ch][For]), and choline acetate ([Ch][Ace]). The prepared solutions were comprised of *D(* +*)*-glucose in water and *D(* +*)*-glucose in aqueous IL solutions over a concentration range of (0.0000 to 0.0900) mol·kg⁻^1^ and at the temperature range of (298.15 to 318.15) K for volumetric, acoustic, and viscosity studies. Conversely, the electrical conductivity measurements of ILs in water and ILs in aqueous *D(* +*)*-glucose solution were examined at a constant temperature of 298.15 K and at the same aforementioned concentration range. Through these measurements some thermodynamic properties such as the apparent molar volume ($V_{\varphi }$) standard partial molar volume ($V_{\varphi }^{0}$), apparent molar isentropic compressibility (*κ*_φ_), partial molar isentropic compressibility ($\kappa_{\varphi }^{0}$), viscosity *B*-coefficients, limiting molar conductivity ($\Lambda_{0}$), and ion association constant (*K*_A_), were computed. The COSMO calculations were employed to provide valuable information such as the sigma profile (*σ*), cavity surface area (*A*), total cavity volume (*V*), dielectric (solvation) energies, and HOMO–LUMO levels. By leveraging these DFT-derived parameters, this approach offers a microscopic perspective that aids in interpreting macroscopic experimental phenomena. The *σ*-profile, in particular, provides critical insights into the electrostatic potential distribution across the molecular framework.

## Experimental measurements

### Materials

The related specification about the chemicals used in this study such as chemical name, chemical formula, provenance, CAS number, molar mass, mass fraction (purity) has been tabulated within Table 1. The utilized water in this study was doubly distilled deionized and had a specific conductivity less than 1 μS∙cm^−1^.

**Table 1:** The specification of the utilized chemicals

Chemical name	Chemical formula	Provenance	CAS.no	Molar mass (g∙mol^−1^)	Mass fraction (purity)
*D(* +*)*-glucose	C_6_H_12_O_6_	Merck	50–99-7	180.16	> 99%
Choline chloride	C_5_H_14_ClNO	Merck	67–48-1	139.62	> 98%
Salicylic acid	C_7_H_6_O_3_	Merck	69–72-7	138.12	> 99%
Formic acid	CH_2_O_2_	Merck	64–18-6	46.03	> 99%
Acetic acid	C_2_H_4_O_2_	Merck	64–19-7	60.050	> 99%
Dichloromethane (DCM)	C_4_H_11_NO_3_	Merck	75–09-2	84.93	> 99%
Methanol	CH_4_O	Merck	67–56-1	32.04	> 98%
Choline Salicylate [Ch][Sal]	C_12_H_19_NO_4_	Synthesized	2016–36-6	241.29	> 82%
Choline Formate [Ch][For]	C_6_H_15_NO_3_	Synthesized	9031–54-3	149.19	> 82%
Choline Acetate [Ch][Ace]	C_7_H_17_NO_3_	Synthesized	14586–35-7	163.22	> 76%

### Synthesis route of the choline- based ILs

#### [Ch][Sal] IL

The synthesis route for the [Ch][Sal] ionic liquid has been provided in Figure S1. The synthesis process of [Ch][Sal] is as follows: an equimolar amount (1:1) of choline chloride (9.2014 g) and sodium salicylate (10.5510 g) were introduced into a 250 mL round-bottom flask. The non-polar solvent dichloromethane (DCM) was employed as a reaction medium, and approximately 80 mL was added to facilitate the reaction kinetics. The reaction vessel was immersed in an oil bath and subjected to vigorous magnetic stirring. The synthesis was conducted at about 298 K under a neutral argon atmosphere for a duration of 72 h to prevent oxidation of the target IL. Upon completion, the crude product was subjected to multiple centrifugation cycles to ensure complete removal of inorganic by-products, namely sodium chloride. Subsequently, the DCM solvent was evaporated under reduced pressure at about 313 K using a rotary evaporator. To further purify the [Ch][Sal] IL, approximately 100 mL of anhydrous DCM was added and the mixture was vigorously agitated. The washing process successfully eliminated residual impurities and inorganic salts, resulting in the formation of a biphasic system. The lower, denser phase enriched in the desired IL was separated from the upper organic phase, which exhibited a turbid appearance due to the presence of impurities. The washing and separation procedure was reiterated until the upper phase attained clarity, indicating the removal of contaminants. The purified IL was finally dried under vacuum at room temperature in an argon-filled desiccator to minimize moisture content, as even trace amounts of water can significantly influence thermophysical properties such as density, viscosity, and electrical conductivity.

#### [Ch][For] and [Ch][Ace] IL

[Ch][For] and [Ch][Ace] were synthesized through a neutralization process (Figs S4 and S7). Initially, choline hydroxide was produced by reacting choline chloride with potassium hydroxide in methanol under reflux conditions. After removing the methanol, the absence of chloride ions was confirmed. The concentration of choline hydroxide was quantified through an acid–base titration using a standardized hydrochloric acid solution. A pH meter (Metrohm, 692 pH/ion meter) was employed to accurately determine the equivalence point of the titration [45]. Subsequently, stoichiometric amounts of formic acid and acetic acid were added to the choline hydroxide solution and stirred at room temperature. The formed water was removed through vacuum distillation, and the crude product was washed with a methanol–acetonitrile mixture to purify the resulted ionic liquids. The final products, [Ch][For] and [Ch][Ace], were obtained after removing the solvents. In order to confirm the purity of the synthesized ionic liquids, the FT-IR (Bruker Tensor 270-KBr) and FT-NMR (Bruker Avance-400 NMR) spectroscopy have been performed.

### Density and speed of sound measurements

Solutions were prepared through utilization of a Shimadzu-AW220 analytical balance with a precision of $\pm 2 \times 10^{ - 4}$ kg. The Density (ρ) and speed of sound (u) data for binary systems of [Ch][Sal], [Ch][For], and [Ch][Ace] in water, as well as ternary systems of the studied ionic liquids in various aqueous glucose solutions, were determined using a DSA 5000 digital densimeter (Anton Paar, Austria) equipped with a high-precision vibrating tube operating at approximately 3 MHz. The densimeter was calibrated using the air/water program, and a built-in Peltier device maintained a constant temperature for all measurements. The estimated standard uncertainties for density and speed of sound measurements were approximately 0.06 × 10⁻^3^ g·cm⁻^3^ and 1 m·s⁻^1^, respectively.

### Viscosity measurement

The viscosity measurements were conducted using an Anton Paar Lovis 2000 M/ME rolling-ball viscometer manufactured in Austria. The instrument's built-in thermostat, employing a Peltier technique, maintained a constant temperature with a precision of ± 0.005 K. The viscometer operates on the falling ball principle, wherein a calibrated glass capillary filled with the sample solution is used to measure the falling time of a steel ball. Kinematic and dynamic viscosities were calculated from the measured falling time and density values. The capillary was pre-calibrated by the manufacturer using viscosity standard fluids. The overall uncertainty in viscosity measurements was determined to be 0.001 mPa·s.

### Electrical conductivity measurements

The electrical conductivity measurements were performed using a Metrohm model 712 conductivity meter that was equipped with a dipping conductivity cell containing platinized electrodes (cell constant: 0.880 cm⁻^1^). The cell constant was determined by calibration with a 0.01 mol·kg⁻^1^ aqueous KCl solution. The conductivity cell was filled with a precisely weighed amount of doubly distilled, deionized, and degassed water containing a known mass of *D(* +*)*-glucose. A defined amount of pure ionic liquid was then injected into the solution and stirred continuously. To ensure temperature stability with a precision of ± 0.02 K, the sample holder was surrounded by a circulating water bath maintained by a Julabo ED thermostat. The estimated uncertainty in measured specific conductivity was less than 0.5%.

## Results and discussion

### ***Characterization of the synthesized Choline based ILs through ***^***1***^***FT-NMR and FT-IR analysis***

FT-NMR and FT-IR spectroscopy are indispensable tools for characterizing materials. The ^1^H-NMR spectra provide detailed structural information, including the number, type, and connectivity of hydrogen atoms within a molecule, enabling precise structural elucidation and purity assessment. FT-IR, on the other hand, identifies functional groups by analyzing the vibrational frequencies of molecular bonds, aiding in qualitative and quantitative analysis, polymer characterization, and surface analysis.

### [Ch][Sal] IL

A Bruker Avance-400 NMR spectrometer was employed for the ^1^H-NMR spectra analysis, with deuterated dimethyl sulfoxide (DMSO) serving as the solvent. The ^1^H-NMR spectrum of [Ch][Sal] IL (Fig S3) provides valuable insights into its molecular structure. The chemical shifts of the protons are influenced by their electronic environment and the presence of neighboring groups. Protons Ha and Hb (δ 1.47 ppm) exhibit distinct chemical shifts due to the shielding effect of the oxygen atom on Ha (δ 6.87 ppm). The chemically equivalent protons H1 and H2 (δ 3.63 ppm) resonate at a similar chemical shift. In contrast, H3 and H4 (δ 3.86 ppm), also chemically equivalent, experience a deshielding effect from the adjacent carbonyl group, resulting in a downfield shift. The methyl protons (δ 1.33 ppm) associated with the choline moiety display a characteristic chemical shift. The detailed analysis of the ^1^H-NMR spectrum confirms the structural features of [Ch][Sal] IL. The observed chemical shifts for each proton type correlate with their expected positions within the molecule and the influence of neighboring functional groups.

The FT-IR spectroscopy (Bruker Tensor 270-KBr) is a valuable technique for elucidating the functional groups present in a molecule. When applied to [Ch][Sal] IL (Fig S2), several key vibrational bands provide structural information. The IR spectrum exhibits a broad absorption band centered around 3500 cm^−1^, characteristic of hydroxyl (O–H) stretching vibrations. This indicates the presence of an alcohol or phenol group, likely associated with the salicylate moiety. Additionally, a sharp peak at 1756 cm^−1^ corresponds to the carbonyl (C = O) stretching vibration, confirming the presence of a carbonyl group within the salicylate structure. Multiple peaks in the region of 1579–63 cm^−1^ are attributed to the aromatic C–C stretching vibrations of the benzene ring, a fundamental component of the salicylate moiety. Furthermore, the IR spectrum depicts absorption bands between 1207 and 1139 cm^−1^, which are probably assigned to C-N stretching vibrations. These bands arise from the nitrogen-containing functional groups present in both the choline and salicylate components of the IL.

### [Ch][For] & [Ch][Ace] IL

The IR spectrum analysis of [Ch][For] (Fig S5) provides valuable insights into its molecular structure. Key functional groups are identified through characteristic absorption bands. A prominent peak at 1594 cm^−1^ confirms the presence of a carbonyl (C = O) group, characteristic of the formate moiety. Additionally, the presence of a carboxylate group (COO^−^) is indicated by a band at 1346 cm^−1^, suggesting the formation of an ionic salt. Further supporting the formate structure by the C-O stretching vibration observed at 1083 cm^−1^. The presence of C-N bending vibrations around 956–65 cm^−1^ suggests the presence of nitrogen-containing functional groups within the choline cation.

The IR spectrum of the [Ch][Ace] IL provides valuable information about its molecular structure. A broad peak centered around 3500 cm⁻^1^ suggests the presence of a hydroxyl (O–H) group. The C-O stretching vibration observed at 1087 cm⁻^1^ confirms the acetate group. Additionally, the C-H bending vibration at 1404 cm⁻^1^ indicates the presence of aliphatic groups. The carbonyl (C = O) stretching vibration is observed at 1670 cm⁻^1^. The C-N stretching vibration at 956 cm⁻^1^ suggests the presence of an amine group, likely associated with the choline cation.

The Bruker Avance-400 NMR spectrometer was employed for the ^1^H-NMR spectra analysis, with deuterated dimethyl sulfoxide (DMSO) serving as the solvent. The ^1^H-NMR spectrum of [Ch][For] IL (Fig S6) provides valuable insights into the chemical environment of its hydrogen atoms. The aldehyde proton (Ha) experiences a significant downfield shift due to the strong electron-withdrawing effect of the adjacent carbonyl group. This places Ha at approximately 9 ppm. The methylene protons (H1 and H2) in the formate moiety resonate at around 3.81 ppm, influenced by both the electron-withdrawing carbonyl and the electron-donating oxygen. The methyl protons (H3 and H4) in the formate group exhibit a slightly lower chemical shift at 3.42 ppm, indicating less influence from the carbonyl group. Finally, the methyl protons of the choline cation appear around 3.13 ppm, shielded by the nitrogen atom.

The methyl protons, influenced by the neighboring oxygen in ^1^H-NMR spectrum analysis of [Ch][Ace] IL (Fig S9), exhibit an unusually low chemical shift at approximately 1.47 ppm. In contrast, the methylene protons (H1 and H2) in the acetate moiety resonate at around 3.83 ppm, influenced by both the electron-withdrawing carbonyl group and the electron-donating oxygen. The methyl protons (H3 and H4) in the acetate group appear at a slightly higher field (3.42 ppm) compared to H1 and H2, indicating a lesser influence from the carbonyl group. Finally, the methyl protons of the choline cation resonate around 2.98 ppm, a typical chemical shift for methyl groups attached to nitrogen. The observed chemical shifts in the ^1^H-NMR spectrum of choline acetate directly correlate with the electronic environment and structural features of the molecule. The anomalous shift of the methyl protons is attributed to the shielding effect of the oxygen atom. The chemical shifts of H1, H2, H3, and H4 are influenced by the interplay between the electron-withdrawing carbonyl group and the electron-donating oxygen atom. The chemical shift of the choline methyl protons is consistent with their position relative to the nitrogen atom.

### Theoretical framework

The theoretical framework relies primarily on the DFT calculation on Dmol3 with COSMO results.

**Fig. 1:**
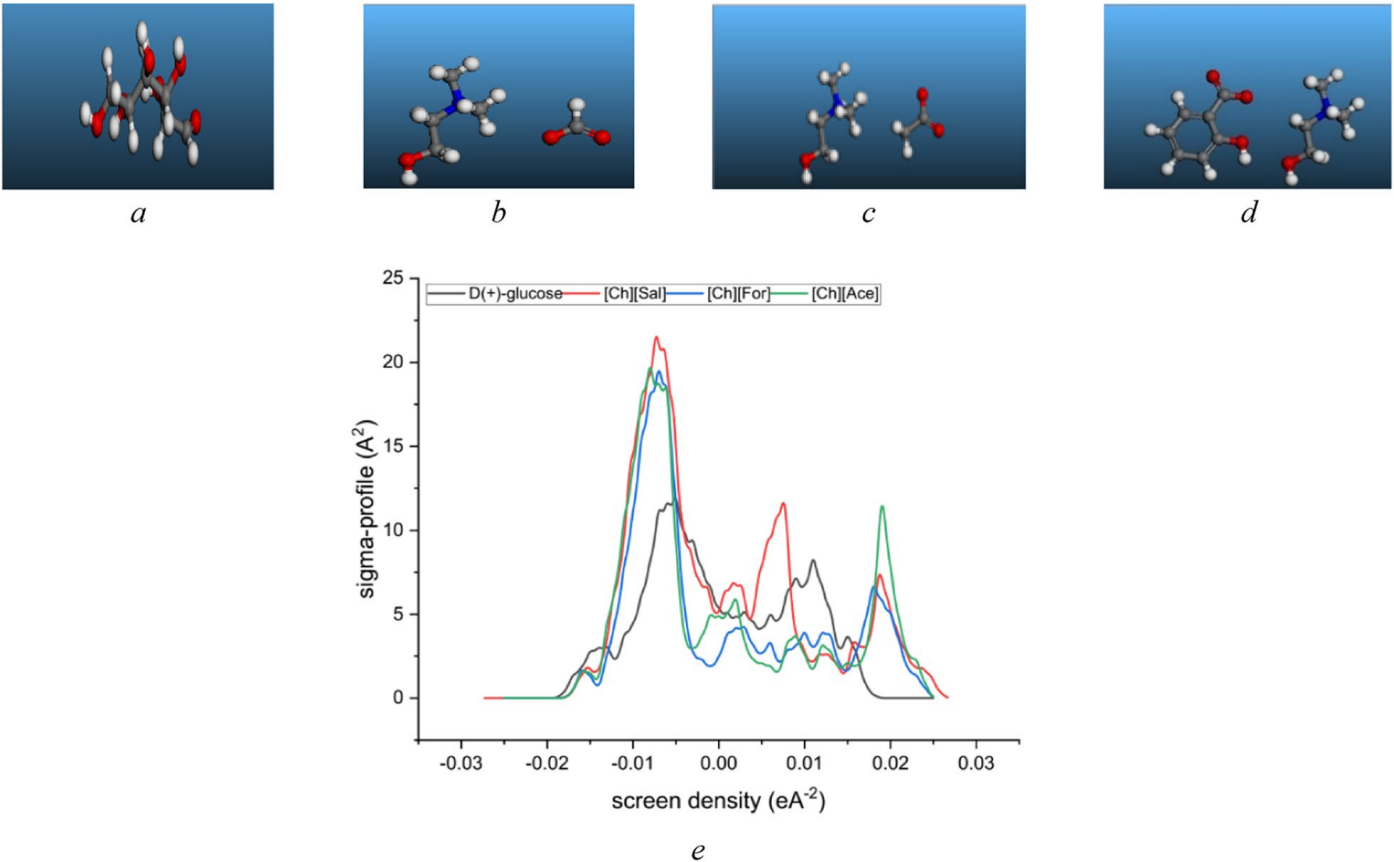
Optimized molecular structure and σ-profile of **a**) *D(* +*)*-glucose, **b** [Ch][For], **c** [Ch][Ace], **d** [Ch][Sal] and **e**) sigma profile plot from Dmol3 and COSMO result

COSMO is a powerful computational chemistry technique used to model the solvation effects of molecules in various solvents. It accurately calculates the solvation energy of a molecule, accounting for electrostatic interactions, hydrogen bonding, and dispersion forces. This information is crucial for understanding solubility, stability, and reactivity in different solvents. Materials Studio (Biovia, 2023) employing the GGA VWN-BP functional was used to achieve the optimal results for the studied system, as recommended by the Dmol3 developers. Also, water was chosen as the solvent for the COSMO calculation. A two-step task including geometry and energy optimization GGA VWN-BP function, DND (3.5) basis set, and COSMO results. The COSMO results containing *σ*-profile illustrated in Fig. 1.

The dielectric energy, a crucial factor influencing hydration behavior has been calculated using DFT-COSMO considered alongside cavity surface area and volume to gain a deeper understanding of the interactions between choline-based ionic liquids and *D*( +)-glucose. The cavity volume, as presented in Table 2 was deemed a representative parameter for the intensity of these interactions [46–48]. The highest cavity volume value observed for [Ch][Sal] suggests that this ionic liquid exhibits the strongest interaction with water, even surpassing that of *D*( +)-glucose. This finding implies that [Ch][Sal] may potentially be the most effective ionic liquid for catalyzing the conversion of *D*( +)-glucose [48–52].

**Table 2:** The surface area (*A*) and total volume of cavity (*V*), dielectric (solvation) energy, HOMO and LUMO values and their respective energies obtained from COSMO and Dmol3 calculations

Chemicals	*A* (A^2^)	*V* (A^3^)	Dielectric (solvation) energy (kcal·mol^−1^)	HOMO	LUMO	*E*_HOMO_ (ev)	*E*_LUMO_ (ev)
*D*( +)-glucose	192.566	184.732	− 28.38	48	49	− 6.030	− 1.981
[Ch][Sal]	283.070	286.072	− 82.19	65	66	− 4.365	− 1.149
[Ch][Ace]	226.286	210.814	− 84.45	45	46	− 4.439	0.327
[Ch][For]	205.790	191.751	− 65.24	41	42	− 4.737	0.348

The core concept in COSMO-based thermodynamics is the *σ*-profile, a molecular fingerprint representing the surface charge distribution. This profile characterizes the probability of specific charge concentrations within defined molecular segments. COSMO models, such as COSMO-RS and COSMO-SAC, leverages the *σ*-profiles to predict thermodynamic properties and intermolecular interactions, providing insights into the interactions between choline based-ILs and *D(* +*)*-glucose relevant to bioethanol catalysis. Typically, σ-profiles for molecules are derived from computationally intensive simulations of molecular electron density using density functional theory (DFT). This DFT-based approach can often be a significant computational bottleneck in theoretical studies [53]. The provided sigma-profiles (Fig. 1) illustrate the charge distribution on the molecular surfaces of choline-based choline based-ILs ([Ch][Sal], [Ch][For], [Ch][Ace]) and *D(* +*)*-glucose. The choline based-ILs exhibit broader and more symmetrical peaks centered around the negative region of the screen density axis, while *D(* +*)*-glucose displays a narrower and more skewed peak with a significant portion extending into the positive region. This indicates a more dispersed charge distribution on the choline based-ILs and a more polar nature for *D(* +*)*-glucose. The negative charge distribution observed in the sigma-profiles of the choline based-ILs suggests a propensity for nucleophilic behavior. Nucleophilic species are known to be attracted to regions of positive charge and are capable of donating electron pairs. *D(* +*)*-glucose, with its polar hydroxyl groups, also possesses nucleophilic sites. However, the presence of a significant portion of its sigma-profile in the positive region indicates that it may also exhibit electrophilic properties. Understanding the nucleophilic behavior of choline-based choline based-ILs and *D(* +*)*-glucose is crucial for comprehending the interactions governing their behavior in aqueous solutions. The nucleophilic nature of the choline based-ILs may contribute to their ability to form hydrogen bonds or other electrostatic interactions with the polar groups of *D(* +*)*-glucose. Additionally, the potential for both nucleophilic and electrophilic behavior in *D(* +*)-glucose* could lead to complex interactions involving both electron donation and acceptance [54].

The properties of choline based-ILs that has been depicted within Table 2 exhibit distinct trends with increasing alkyl chain length. The dielectric solvation energy, a measure of the interaction between the IL and its solvent (water in this case), becomes progressively more negative from choline formate to choline acetate and then to choline salicylate. This suggests that the longer alkyl chain enhances the solvation process. As the chain length of the choline based-ILs increases, both the energy levels of the HOMO and the LUMO become less negative. This trend suggests a decrease in the ionic liquids ability to donate or accept electrons. This change in electronic properties may influence the interactions between the ionic liquids and glucose molecules in solution. Consequently, the *E*_HOMO_ values follow a similar trend, reflecting the energy of the HOMO. In contrast, E_LUMO_ values demonstrate a more complex behavior, transitioning from positive to negative as the chain length increases. These findings suggest that the elongation of the alkyl chain in choline-based choline based-ILs influences their electronic structure and solvent interactions. The increased negative dielectric solvation energy implies stronger solvent interactions, while the changes in HOMO and LUMO energies suggest alterations in the molecule's ability to participate in electron transfer processes.

### Volumetric properties

**Fig. 2:**
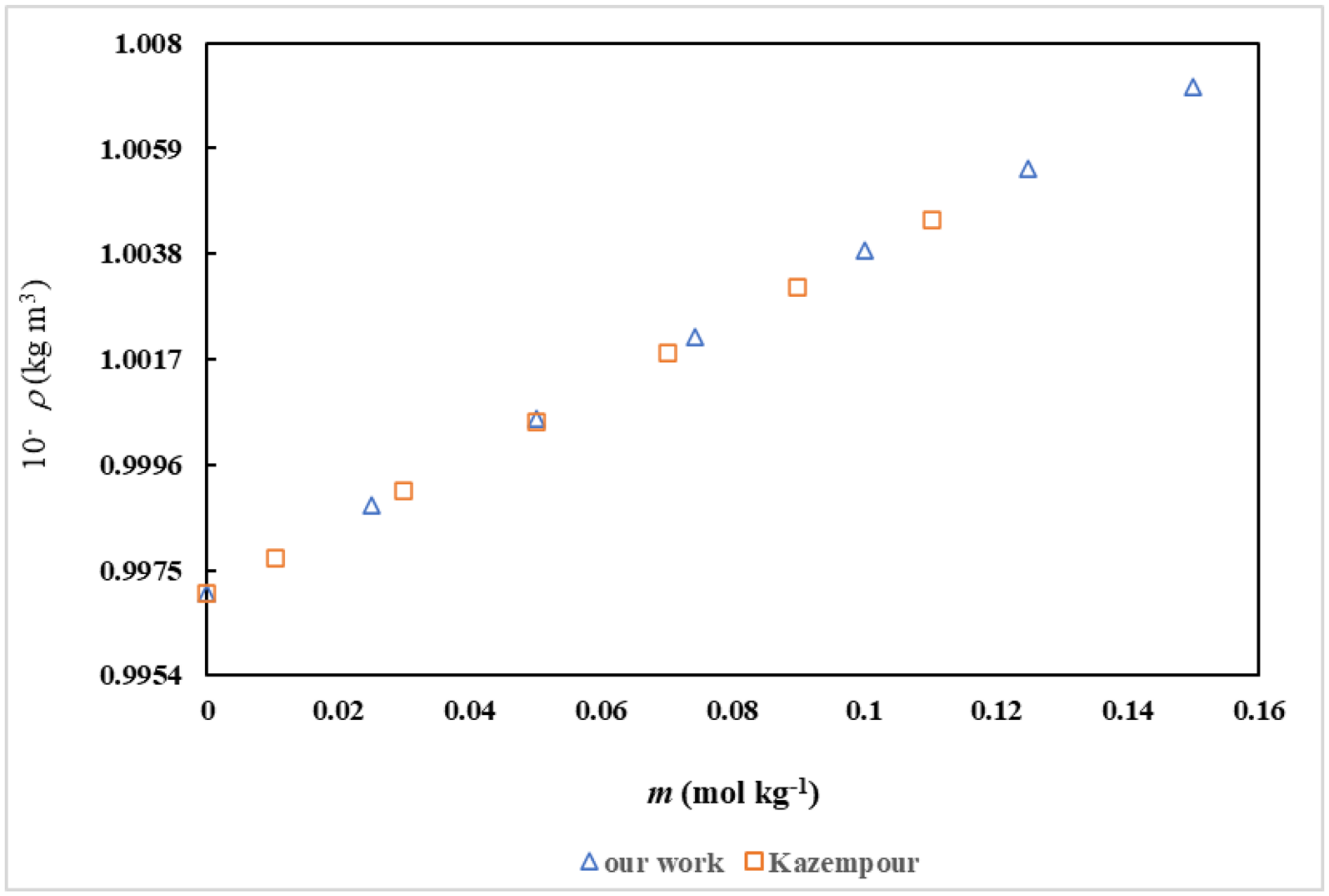
Comparison of the density of D( +)-glucose in water at 298.15: triangle up open our work (Δ), box open from ref [55] (⊡)

The density (ρ) values of *D*( +)-glucose in water, measured in this study, have been validated by comparison with literature data, as presented in Fig. 2.

Furthermore, the density values of aqueous solutions containing 0.05, 0.10, and 0.15 mol∙kg⁻^1^ of the choline-based ionic liquids [Ch][Sal], [Ch][For], and [Ch][Ace] have been systematically recorded in Table 3.

**Table 3:** The density data ($\rho$)*,* and apparent molar volume ($V_{\varphi }$), values for *D(* +*)*-glucose in the aqueous ILs solutions at various temperatures^*a*^

*m* (mol·kg^−1^)	10^–3^ *ρ* (kg·m^−3^)					10^6^ *Vφ* (m^3^·mol^−1^)				
*T* (K)	298.15	303.15	308.15	313.15	318.15	298.15	303.15	308.15	313.15	318.15
*D(* +*)*-glucose in water										
0.0000	0.997042	0.995646	0.994023	0.992208	0.990201					
0.0250	0.998792	0.997390	0.995761	0.993941	0.991931	110.08	110.38	110.68	110.96	111.17
0.0500	1.000510	0.999106	0.997473	0.995648	0.993633	110.52	110.74	111.01	111.29	111.53
0.0741	1.002144	1.000741	0.999107	0.997272	0.995251	110.89	111.04	111.26	111.61	111.89
0.1001	1.003885	1.002466	1.000827	0.998992	0.996965	111.15	111.44	111.67	111.95	112.24
0.1249	1.005510	1.004084	1.002438	1.000603	0.998586	111.54	111.84	112.10	112.34	112.51
0.1498	1.007116	1.005699	1.004058	1.002213	1.000186	111.92	112.12	112.31	112.60	112.82
*D(* +*)*-glucose in aqueous solution of [Ch][Sal] (0.0299 mol.kg^−1^)										
0.0000	0.998327	0.996908	0.995268	0.993431	0.991394					
0.0252	1.000047	0.998623	0.996976	0.995132	0.993092	111.81	112.07	112.42	112.78	112.99
0.0503	1.001747	1.000317	0.998661	0.996815	0.994765	111.85	112.13	112.52	112.79	113.14
0.0750	1.003413	1.001967	1.000301	0.998450	0.996405	111.82	112.25	112.67	112.94	113.14
0.1003	1.005100	1.003649	1.001986	1.000126	0.998073	111.93	112.32	112.62	112.94	113.19
0.1247	1.006723	1.005268	1.003583	1.001717	0.999663	111.97	112.32	112.76	113.08	113.31
0.1500	1.008393	1.006933	1.005238	1.003360	1.001304	111.99	112.33	112.78	113.14	113.36
*D(* +*)*-glucose in aqueous solution of [Ch][Sal] (0.0596 mol.kg^−1^)										
0.0000	0.999602	0.998161	0.996500	0.994644	0.992616					
0.0250	1.001305	0.999857	0.998191	0.996329	0.994295	111.87	112.21	112.49	112.81	113.15
0.0500	1.002995	1.001537	0.999865	0.997995	0.995950	111.88	112.29	112.58	112.95	113.39
0.0750	1.004676	1.003212	1.001532	0.999654	0.997600	111.93	112.31	112.64	113.02	113.46
0.0999	1.006340	1.004860	1.003173	1.001289	0.999235	111.96	112.42	112.76	113.12	113.48
0.1248	1.007995	1.006505	1.004810	1.002920	1.000854	111.98	112.44	112.79	113.15	113.55
0.1499	1.009650	1.008155	1.006449	1.004545	1.002460	112.03	112.46	112.84	113.25	113.73
*D(* +*)*-glucose in aqueous solution of [Ch][Sal] (0.0894 mol.kg^−1^)										
0.0000	1.000832	0.999370	0.997675	0.995815	0.993759					
0.0250	1.002532	1.001064	0.999363	0.997496	0.995434	111.94	112.26	112.59	112.95	113.31
0.0491	1.004157	1.002681	1.000974	0.999100	0.997029	111.99	112.33	112.65	113.02	113.40
0.0745	1.005864	1.004380	1.002665	1.000785	0.998705	112.05	112.39	112.73	113.10	113.50
0.0999	1.007551	1.006063	1.004340	1.002450	1.000364	112.10	112.45	112.80	113.17	113.59
0.1244	1.009170	1.007672	1.005943	1.004050	1.001951	112.16	112.52	112.87	113.24	113.68
0.1499	1.010845	1.009338	1.007604	1.005699	1.003591	112.21	112.58	112.94	113.31	113.77
*D(* +*)*-glucose in aqueous solution of [Ch][For] (0.0293 mol.kg^−1^)										
0.0000	0.997369	0.996046	0.994746	0.993404	0.992020					
0.0251	0.999120	0.997784	0.996473	0.995121	0.993727	110.26	110.83	111.33	111.79	112.25
0.0504	1.000865	0.999519	0.998189	0.996831	0.995433	110.49	111.01	111.66	112.04	112.39
0.0752	1.002550	1.001181	0.999855	0.998483	0.997079	110.83	111.50	111.91	112.37	112.71
0.1003	1.004226	1.002849	1.001507	1.000135	0.998721	111.13	111.73	112.21	112.58	112.94
0.1252	1.005884	1.004486	1.003136	1.001744	1.000330	111.30	111.96	112.42	112.89	113.20
0.1492	1.007475	1.006093	1.004732	1.003340	1.001916	111.74	112.20	112.67	113.07	113.41
*D(* +*)*-glucose in aqueous solution of [Ch][For] (0.0603 mol.kg^−1^)										
0.0000	0.997845	0.996530	0.995291	0.994070	0.992931	–	–	–	–	
0.0252	0.999573	0.998250	0.997003	0.995774	0.994628	111.45	111.83	112.20	112.58	112.91
0.0501	1.001267	0.999936	0.998678	0.997445	0.996292	111.52	111.90	112.34	112.64	112.97
0.0751	1.002949	1.001615	1.000350	0.999104	0.997944	111.71	112.02	112.43	112.82	113.16
0.1001	1.004619	1.003272	1.001999	1.000747	0.999581	111.82	112.20	112.60	112.97	113.30
0.1248	1.006258	1.004902	1.003624	1.002356	1.001186	111.93	112.32	112.69	113.13	113.43
0.1502	1.007915	1.006552	1.005261	1.004000	1.002829	112.10	112.48	112.89	113.22	113.49
*D(* +*)*-glucose in aqueous solution of [Ch][For] (0.0900 mol.kg^−1^)										
0.0000	0.998329	0.997008	0.995740	0.994514	0.993349	–	–	–	–	
0.0253	1.000050	0.998722	0.997447	0.996212	0.995040	111.94	112.28	112.62	113.03	113.36
0.0498	1.001709	1.000375	0.999093	0.997848	0.996668	111.94	112.26	112.60	113.04	113.40
0.0747	1.003386	1.002045	1.000755	0.999507	0.998319	112.01	112.33	112.69	113.04	113.41
0.0999	1.005077	1.003726	1.002426	1.001162	0.999963	111.90	112.26	112.64	113.08	113.48
0.1245	1.006713	1.005352	1.004044	1.002788	1.001579	111.98	112.36	112.75	113.05	113.46
0.1497	1.008366	1.007004	1.005691	1.004409	1.003215	112.07	112.40	112.76	113.20	113.45
*D(* +*)*-glucose in aqueous solution of [Ch][Ace] (0.0310 mol.kg^−1^)										
0.0000	0.997242	0.995940	0.994669	0.993468	0.992311					
0.0254	0.998972	0.997665	0.996390	0.995186	0.994027	111.96	112.22	112.43	112.60	112.73
0.0499	1.000629	0.999317	0.998038	0.996832	0.995670	112.01	112.27	112.49	112.65	112.80
0.0752	1.002329	1.001012	0.999729	0.998519	0.997356	112.07	112.32	112.54	112.72	112.85
0.1000	1.003985	1.002664	1.001376	1.000164	0.998998	112.12	112.37	112.60	112.77	112.91
0.1251	1.005647	1.004323	1.003030	1.001812	1.000647	112.18	112.41	112.65	112.84	112.96
0.1500	1.007280	1.005951	1.004656	1.003432	1.002265	112.23	112.47	112.69	112.91	113.03
*D(* +*)*-glucose in aqueous solution of [Ch][Ace] (0.0601 mol.kg^−1^)										
0.0000	0.997588	0.996270	0.995002	0.993807	0.992664					
0.0257	0.999337	0.998015	0.996742	0.995543	0.994395	111.98	112.20	112.45	112.66	112.91
0.0499	1.000974	0.999645	0.998371	0.997165	0.996016	112.03	112.31	112.49	112.76	112.94
0.0750	1.002656	1.001321	1.000044	0.998833	0.997683	112.09	112.38	112.55	112.82	112.97
0.0997	1.004304	1.002965	1.001684	1.000469	0.999317	112.15	112.42	112.61	112.87	113.01
0.1248	1.005965	1.004622	1.003336	1.002118	1.000962	112.19	112.45	112.65	112.90	113.05
0.1500	1.007624	1.006278	1.004986	1.003768	1.002605	112.24	112.49	112.71	112.92	113.11
*D(* +*)*-glucose in aqueous solution of [Ch][Ace] (0.0898 mol.kg^−1^)										
0.0000	0.997987	0.996670	0.995415	0.994214	0.993067					
0.0250	0.999684	0.998361	0.997101	0.995895	0.994744	112.17	112.47	112.73	112.99	113.20
0.0502	1.001387	1.000055	0.998791	0.997580	0.996420	112.17	112.53	112.76	113.02	113.33
0.0750	1.003051	1.001712	1.000443	0.999228	0.998064	112.20	112.55	112.80	113.04	113.32
0.1000	1.004717	1.003369	1.002096	1.000878	0.999707	112.22	112.59	112.83	113.06	113.35
0.1249	1.006367	1.005012	1.003733	1.002510	1.001337	112.24	112.61	112.86	113.10	113.36
0.1496	1.007990	1.006627	1.005343	1.004118	1.002934	112.27	112.64	112.89	113.11	113.42

^a^The standard uncertainties for molality, temperature and pressure were* u* (*m*) = 0.001 mol kg^−1^, *u* (*T*) = 0.2 K, *u* (*P*) = 10.5 hPa, respectively with level of confidence 0.95. The standard combined uncertainty for density and apparent molar volume were about, *u*_*c*_ (*ρ*) = 0.06 × 10^–3^ g cm^−3^ and *u*_*c*_(*V*_*φ*_) = 5 × 10^–5^ m^3^ mol^−1^ (level of confidence 0.68), respectively

**Fig. 3:**
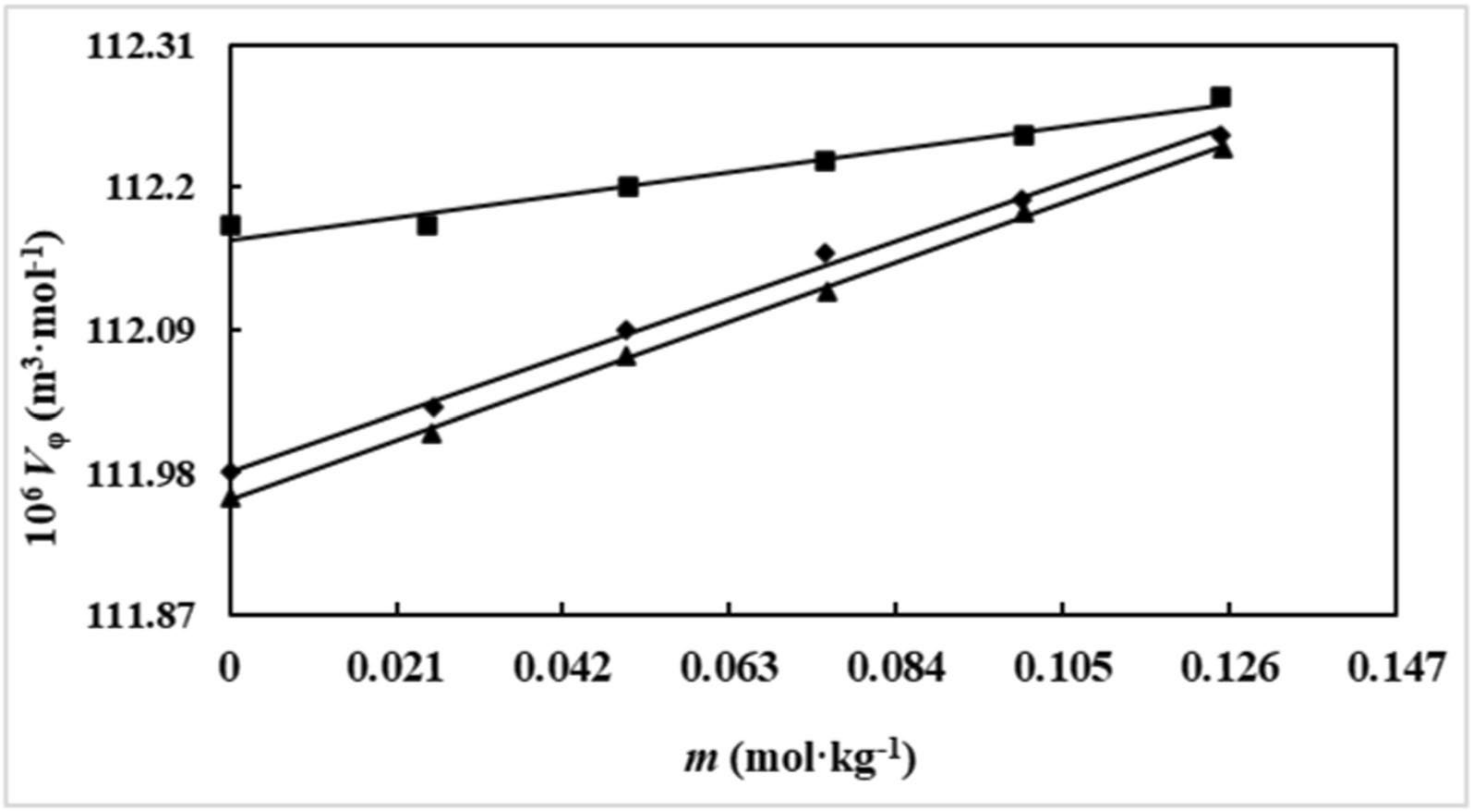
The apparent molar volumes ($V_{\varphi }$/ m^3^.mol^−1^) of *D(* +*)*-glucose versus its molality (*m* / mol.kg^−1^) in aqueous [Ch][Ace] solutions with varying concentrations:∎, 0.0900; ◆, 0.0600; ▲, 0.0298 (mol.kg^−1^) at *T* = 298.15 K

An analysis of Table 3 reveals several trends. Firstly, the density of the solutions increases with increasing *D(* +*)*-glucose concentration. This behavior is expected due to the inherent denser nature of *D(* +*)*-glucose compared to water. As the proportion of *D(* +*)*-glucose in the solution increases, the overall density of the mixture rises. Secondly, the density is observed to be higher for ionic liquids with longer alkyl chains. For example, [Ch][Sal] with the longest alkyl chain exhibits higher density compared to [Ch][For] and [Ch][Ace]. This can be attributed to the increased van der Waals interactions between the longer alkyl chains of the ionic liquids, leading to a more tightly packed structure and consequently, higher density. Finally, the density of the solutions displays opposing trends with respect to temperature and ionic liquid content. Increasing the temperature generally leads to a decrease in solution density due to the thermal expansion of the solvent molecules. Conversely, increasing the IL content in the solutions often results in a density increase, as ionic liquids typically possess higher densities than water. The apparent molar volumes ($V_{\varphi }$) of *D(* +*)*-glucose in the examined solutions were calculated using the following expression [56]:

1 $$V_{\varphi } = \frac{M}{\rho } - \frac{{(\rho - \rho_{0} )}}{{m\rho \rho_{0} }}$$

where *M* is the *D(* +*)*-glucose molar mass, *m* is the molality of *D(* +*)*-glucose in aqueous ionic liquids solutions, and $\rho$, and $\rho_{0}$ represents the densities of *D(* +*)*-glucose in aqueous IL and ionic liquids in water solutions, respectively. Table 3, also depicts the derived values of $V_{\varphi }$ for *D(* +*)*-glucose in water and aqueous ionic liquids solution across a temperature range of (298.15 to 318.15) K, with intervals of 5 K. The variation of $V_{\varphi }$ values of *D(* +*)*-glucose in aqueous [Ch][Ace] solutions have been graphically represented in Fig. 3.

The values of $V_{\varphi }$, within the studied temperature range, exhibit an increasing trend with rising the IL content. A robust linear correlation was observed between $V_{\varphi }$ values and *D(* +*)*-glucose molality (*m*). Similar behavior was also noted for $\rho$. Consequently, standard partial molar volumes ($V_{\varphi }^{0}$) values were determined by applying least-squares fitting to Masson's equation [56]:

2 $$V_{\varphi } = V_{\varphi }^{0} + S_{v} m$$

where *S*_*v*_ is the empirical parameters. The standard partial molar volumes $V_{\varphi }^{0}$ provide valuable insights into solute–solvent interactions as solute–solute interactions become negligible at infinite dilution. The values of $V_{\varphi }^{0}$, *S*_*v*_ together with their standards deviation of the $V_{\varphi }^{0}$ values have been reported in Table 4.

**Table 4:** The standard partial molar volumes ($V_{\varphi }^{0}$), experimental parameter of $S_{v}$, transfer volume ($\Delta_{tr} V_{\varphi }^{0}$), and standard deviations ($\sigma ( V_{\varphi }^{0} )$) for *D(* +*)*-glucose in aqueous solutions of choline-based ILs at different temperatures^a^

*T* (K)	10^6^ *S*_v_ (m^3^·kg·mol^−2^)	10^6^ *V*_φ_^0^ (m^3^·mol^−1^)	10^6^ Δ_tr_*V*_φ_^0^ (m^3^·mol^−1^)	*σ* (V^0^_φ_)
*D(* +*)*-glucose in water				
298.15	14.29 ± 0.479	109.77 ± 0.047	–	0.04
		111.67 [55]		
		111.08 [57]		
		111.91 [58]		
303.15	14.21 ± 0.315	110.02 ± 0.031	–	0.03
308.15	13.54 ± 0.570	110.32 ± 0.055	–	0.05
313.15	13.38 ± 0.282	110.62 ± 0.027	–	0.02
318.15	13.26 ± 0.370	110.87 ± 0.036	–	0.03
*D(* +*)*-glucose in aqueous solutions of [Ch][Sal] (0.0298 mol.kg^−1^)				
298.15	1.61 ± 0.280	111.75 ± 0.027	1.99	0.02
303.15	2.26 ± 0.416	112.04 ± 0.041	2.02	0.04
308.15	2.82 ± 0.490	112.38 ± 0.048	2.06	0.04
313.15	3.06 ± 0.398	112.68 ± 0.039	2.05	0.03
318.15	2.77 ± 0.336	112.95 ± 0.033	2.08	0.03
*D(* +*)*-glucose in aqueous solutions of [Ch][Sal] (0.0600 mol.kg^−1^)				
298.15	1.29 ± 0.105	111.83 ± 0.010	2.06	0.01
303.15	2.06 ± 0.257	112.17 ± 0.025	2.15	0.02
308.15	2.85 ± 0.237	112.43 ± 0.023	2.11	0.02
313.15	3.31 ± 0.284	112.76 ± 0.028	2.14	0.02
318.15	3.94 ± 0.631	113.12 ± 0.061	2.24	0.05
*D(* +*)*-glucose in aqueous solutions of [Ch][Sal] (0.0900 mol.kg^−1^)				
298.15	2.19 ± 0.115	111.89 ± 0.011	2.12	0.01
303.15	2.53 ± 0.131	112.20 ± 0.013	2.18	0.01
308.15	2.79 ± 0.170	112.52 ± 0.016	2.19	0.02
313.15	2.91 ± 0.140	112.88 ± 0.014	2.26	0.01
318.15	3.67 ± 0.298	113.22 ± 0.029	2.35	0.03
*D(* +*)*-glucose in aqueous solutions of [Ch][For] (*0.0298* mol.kg^−1^)				
298.15	11.632 ± 0.605	109.940 ± 0.059	0.170	0.052
303.15	11.380 ± 0.798	110.543 ± 0.078	0.523	0.068
308.15	10.656 ± 0.337	111.102 ± 0.033	0.782	0.029
313.15	10.497 ± 0.370	111.538 ± 0.036	0.918	0.032
318.15	9.679 ± 0.409	111.968 ± 0.040	1.098	0.035
*D(* +*)*-glucose in aqueous solutions of [Ch][For] (0.0600 mol.kg^−1^)				
298.15	5.221 ± 0.263	111.299 ± 0.026	1.529	0.022
303.15	5.373 ± 0.292	111.656 ± 0.028	1.636	0.025
308.15	5.332 ± 0.279	112.059 ± 0.027	1.739	0.024
313.15	5.514 ± 0.332	112.409 ± 0.032	1.789	0.028
318.15	5.044 ± 0.380	112.768 ± 0.037	1.898	0.032
*D(* +*)*-glucose in aqueous solutions of [Ch][For] (0.0900 mol.kg^−1^)				
298.15	0.713 ± 0.520	111.911 ± 0.051	2.141	0.044
303.15	0.962 ± 0.411	112.233 ± 0.040	2.213	0.035
308.15	1.290 ± 0.343	112.564 ± 0.033	2.244	0.029
313.15	1.029 ± 0.455	112.985 ± 0.044	2.365	0.039
318.15	0.781 ± 0.259	113.360 ± 0.025	2.490	0.022
*D(* +*)*-glucose in aqueous solutions of [Ch][Ace] (0.03 mol.kg^−1^)				
298.15	2.192 ± 0.189	111.937 ± 0.018	2.136	0.001
303.15	2.017 ± 0.052	112.168 ± 0.005	2.149	0.004
308.15	2.116 ± 0.052	112.382 ± 0.005	2.058	0.004
313.15	2.451 ± 0.084	112.532 ± 0.008	1.908	0.007
318.15	2.279 ± 0.049	112.68 ± 0.004	1.809	0.004
*D(* +*)*-glucose in aqueous solutions of [Ch][Ace] (0.06 mol.kg^−1^)				
298.15	2.11 ± 0.179	112.04 ± 0.017	2.161	0.004
303.15	2.22 ± 0.285	112.18 ± 0.028	2.161	0.024
308.15	2.13 ± 0.068	112.39 ± 0.006	2.066	0.005
313.15	1.99 ± 0.280	112.65 ± 0.027	2.023	0.024
318.15	1.59 ± 0.093	112.86 ± 0.009	1.987	0.007
*D(* +*)*-glucose in aqueous solutions of [Ch][Ace] (0.09 mol.kg^−1^)				
298.15	0.832 ± 0.425	112.272 ± 0.041	2.371	0.009
303.15	1.283 ± 0.085	112.452 ± 0.008	2.433	0.007
308.15	1.301 ± 0.012	112.698 ± 0.001	2.374	0.001
313.15	0.988 ± 0.049	112.965 ± 0.004	2.341	0.004
318.15	1.354 ± 0.347	113.213 ± 0.034	2.342	0.03

^a^The standard uncertainties for molality, temperature and pressure were* u* (*m*) = 0.001 mol kg^−1^, *u* (*T*) = 0.2 K, *u* (*P*) = 10.5 hPa, respectively with level of confidence 0.95

It is noteworthy that all $V_{\varphi }^{0}$ values, indicative of solute–solvent interactions, are positive and exhibit an increasing trend with both elevated IL content and temperature. This behavior can be attributed to reduced electrostriction of water and intensified solute–solvent interactions. The observed enhancement of $V_{\varphi }^{0}$ at higher temperatures likely stems from the liberation of solvent molecules into the bulk. Similarly, the larger values obtained for ternary systems suggest a comparable phenomenon.$V_{\varphi }^{0}$ values temperature dependency can be expressed by following formula [59]:

3 $$V_{\varphi }^{0} = A + BT + CT^{2}$$

Here *A, B* and *C* are empirical constants which are calculated by the least-square fitting of $V_{\varphi }^{0}$ at investigated temperatures. Standard apparent molar expansibilities $E_{\varphi }^{0}$ were computed from the temperature derivative of $V_{\varphi }^{0}$ at constant pressure, as derived from Eq. 3. The resulting values are presented in Table 5. The calculated $E_{\varphi }^{0}$ values for *D(* +*)*-glucose in aqueous IL solutions are positive. This positive expansibility is characteristic of solutions exhibiting hydrophobic hydration. Consequently, the solution volume increases at a faster rate than pure water, leading to positive $E_{\varphi }^{0}$ values. This phenomenon has been extensively studied in the literature. The $E_{\varphi }^{0}$ values are positive and exhibit an increasing trend with both elevated IL concentration and temperature. This suggests that the systems are temperature-sensitive, with enhanced molecular mobility at higher temperatures. Also, through $E_{\varphi }^{0}$ and $V_{\varphi }^{0}$ values, one can obtain the thermal expansion coefficient, $\alpha$, by utilizing Eq. (4) [59]:

4 $$\alpha = \frac{{E_{\varphi }^{0} }}{{V_{\varphi }^{0} }}$$

**Table 5:** The standard apparent molar expansibility ($E_{\varphi }^{0}$), thermal expansion coefficient ($\alpha$), Hepler's constant $(\partial^{2} V_{\varphi }^{0} /\partial T^{2} )_{P}$ of *D(* +*)*-glucose in water and in the aqueous ionic liquids solutions at *T* = (298.15 to 318.15) K and under atmospheric pressure^a^

*T/*K	*E*_φ_^0^ (m^3^·mol^−1^·K^−1^)	10^3^ $\alpha ($ K^−1^)	10^2^ $(\partial^{2} V_{\varphi }^{0} /\partial T^{2} )_{P}$
*D(* +*)*-glucose in water			
298.15	0.057	5.20	− 0.0200
303.15	0.057	5.15	
308.15	0.056	5.10	
313.15	0.056	5.05	
318.15	0.055	4.99	
*D(* +*)*-glucose in aqueous solutions of [Ch][Sal] (0.0298 mol.kg^−1^)			
298.15	0.065	5.81	− 4.2600
303.15	0.063	5.60	
308.15	0.061	5.40	
313.15	0.058	5.19	
318.15	0.056	4.99	
*D(* +*)*-glucose in aqueous solutions of [Ch][Sal] (0.0606 mol.kg^−1^)			
298.15	0.058	5.18	5.3000
303.15	0.061	5.40	
308.15	0.063	5.63	
313.15	0.066	5.84	
318.15	0.069	6.06	
*D(* +*)*-glucose in aqueous solutions of [Ch][Sal] (0.0901 mol.kg^−1^)			
298.15	0.061	5.48	5.7000
303.15	0.064	5.72	
308.15	0.067	5.96	
313.15	0.070	6.19	
318.15	0.073	6.42	
*D(* +*)*-glucose in aqueous solutions of [Ch][For] (0.0305 mol.kg^−1^)			
298.15	0.118	10.7	− 0.0010
303.15	0.109	9.89	
308.15	0.101	9.092	
313.15	0.093	8.311	
318.15	0.084	7.536	
*D(* +*)*-glucose in aqueous solutions of [Ch][For] (0.0595 mol.kg^−1^)			
298.15	0.077	6.881	− 0.0002
303.15	0.075	6.735	
308.15	0.074	6.588	
313.15	0.072	6.445	
318.15	0.071	6.302	
*D(* +*)*-glucose in aqueous solutions of [Ch][For] (0.0895 mol.kg^−1^)			
298.15	0.062	5.522	0.0011
303.15	0.067	6.006	
308.15	0.073	6.487	
313.15	0.079	6.959	
318.15	0.084	7.431	
*D(* +*)*-glucose in aqueous solutions of [Ch][Ace] (0.0298 mol.kg^−1^)			
298.15	0.059	5.302	− 0.0021
303.15	0.049	4.355	
308.15	0.038	3.414	
313.15	0.028	2.477	
318.15	0.017	1.543	
*D(* +*)*-glucose in aqueous solutions of [Ch][Ace] (0.0599 mol.kg^−1^)			
298.15	0.048	4.328	− 0.0002
303.15	0.047	4.233	
308.15	0.047	4.140	
313.15	0.046	4.046	
318.15	0.045	3.954	
*D(* +*)*-glucose in aqueous solutions of [Ch][Ace] (0.0897 mol.kg^−1^)			
298.15	0.060	5.319	− 0.0006
303.15	0.056	5.018	
308.15	0.053	4.723	
313.15	0.05	4.427	
318.15	0.047	4.134	

^a^The standard uncertainties for molality, temperature and pressure were* u* (*m*) = 0.001 mol kg^−1^, *u* (*T*) = 0.2 K, *u* (*P*) = 10.5 hPa, respectively with level of confidence 0.68

Table 5 presents the $\alpha$ values for the investigated systems. This parameter serves as a quantitative measure of the solutions' response to temperature fluctuations.

The second derivative of $V_{\varphi }^{0}$ with respect to temperature is often called Hepler’s constant and is a representative of the structure breaker or maker behavior of *D(* +*)*-glucose in the presence of aqueous Ionic liquids solutions [59]:

5 $$\left( {{\raise0.7ex\hbox{${\partial E_{\varphi }^{0} }$} \!\mathord{\left/ {\vphantom {{\partial E_{\varphi }^{0} } {\partial T}}}\right.\kern-0pt} \!\lower0.7ex\hbox{${\partial T}$}}} \right)_{P} = \left( {{\raise0.7ex\hbox{${\partial^{2} V_{\varphi }^{0} }$} \!\mathord{\left/ {\vphantom {{\partial^{2} V_{\varphi }^{0} } {\partial T^{2} }}}\right.\kern-0pt} \!\lower0.7ex\hbox{${\partial T^{2} }$}}} \right)_{p} = 2C$$

The Hepler's constants for the investigated systems have been tabulated within Table 5. Negative Hepler's constant values indicate structure-breaking behavior of *D(* +*)*-glucose in aqueous IL solutions, while positive values suggest structure-making behavior [40]. It is noteworthy that the Hepler's constants for *D(* +*)*-glucose in pure water is less negative and approximately near zero, suggesting that *D(* +*)*-glucose shows structure-making behavior from itself in the presence of water. To eliminate the influence of solute–solute and solvent–solvent interactions, transfer volumes ($\Delta_{tr} V_{\varphi }^{0}$) of *D(* +*)*-glucose from water to aqueous IL solutions were calculated at infinite dilution using the following equation [59]:

6 $$\Delta_{tr} V_{\varphi }^{0} = \,V_{\varphi }^{0} \left( {D(\, + ) - {\text{glucose in aqueous ILs}}} \right)\, - V_{\varphi }^{0} \left( {D(\, + ) - {\text{glucose in water}}} \right)$$

Table 4 presents the computed $\Delta_{tr} V_{\varphi }^{0}$ values at infinite dilution. The $\Delta_{tr} V_{\varphi }^{0}$ values are unequivocally positive and increase with rising Ionic liquids molality. In accordance with the co-sphere overlap model for ternary mixtures, interactions between co-sphere and IL species in water can be categorized into: (a) hydrophilic-ionic, (b) hydrophilic-hydrophilic, (c) hydrophilic-hydrophobic, and (d) hydrophobic-hydrophobic [60–62]. Based on this model, interactions (a) and (b) contribute to positive $\Delta_{tr} V_{\varphi }^{0}$ values, while (c) and (d) result in negative values. The observed positive $\Delta_{tr} V_{\varphi }^{0}$ values suggest that hydrophilic interactions between co-sphere molecules and IL ions or polar groups predominate. Furthermore, the increasing trend in $\Delta_{tr} V_{\varphi }^{0}$ at higher IL concentrations indicates intensified interactions of this type. Consequently, a complex interplay of interactions between the solute (*D(* +*)*-glucose) and co-solvent (IL) species is evident.

### Acoustic properties

The apparent molar isentropic compressibility ($\kappa_{\varphi }$) for *D(* +*)*-glucose in aqueous IL solutions at different temperatures was determined using the following equation [63]:

7 $$\kappa_{\varphi } = (\frac{{M\kappa_{s} }}{\rho }) - \left[ {\frac{{\kappa_{s.0} \rho - \kappa_{s} \rho_{0} }}{{m\rho \rho_{0} }}} \right]$$

where *m* is the molality of *D(* +*)*-glucose in the aqueous IL solution, *M* is the molar mass of *D(* +*)*-glucose, $\rho$, is the density of the solutions containing *D(* +*)*-glucose in aqueous ILs solutions and $\rho_{0}$ is the density of ILs in water, respectively. The isentropic compressibility’s of the pure solvent $\kappa_{s.0}$ and solution $\kappa_{s}$ were calculated using the following formula [63]:

8 $$\kappa_{s} = \frac{1}{{u^{2} \rho }}$$

Here *u* (as provided in Table 6) and *ρ*, represent the speed of sound and density of the studied solutions, respectively. The resulting $\kappa_{\varphi }$ values for *D(* +*)*-glucose in aqueous IL solutions (at 0.05, 0.10, and 0.15 mol∙kg⁻^1^) across the experimental temperatures are presented in Table 6.

**Table 6:** The values of speed of sound, *u*, and apparent molar isentropic compressibility, $\kappa_{\varphi }$, for *D(* +*)*-glucose in the aqueous ILs solutions at different temperature and *P* = 0.0871 MPa. ^*a*^

*m* (mol·kg^−1^)	*u* (m·s^−1^)					10^14^ *κ*_φ_ (m^3^·mol^−1^·Pa^−1^)				
*T* (K)	298.15	303.15	308.15	313.15	318.15	298.15	303.15	308.15	313.15	318.15
*D(* +*)*-glucose in water										
0.0000	1496.96	1509.44	1520.15	1529.23	1536.73	–	–	–	–	–
0.0250	1498.42	1510.90	1521.61	1530.69	1538.19	− 1.73	− 1.66	− 1.61	− 1.57	− 1.55
0.0500	1499.90	1512.38	1523.09	1532.17	1539.67	− 1.71	− 1.65	− 1.60	− 1.56	− 1.53
0.0741	1501.35	1513.83	1524.54	1533.62	1541.12	− 1.69	− 1.64	− 1.60	− 1.55	− 1.52
0.1001	1502.91	1515.39	1526.10	1535.18	1542.68	− 1.68	− 1.61	− 1.57	− 1.53	− 1.50
0.1249	1504.45	1516.93	1527.64	1536.72	1544.22	− 1.67	− 1.60	− 1.55	− 1.52	− 1.50
0.1498	1506.01	1518.49	1529.20	1538.28	1545.78	− 1.65	− 1.60	− 1.55	− 1.51	− 1.49
D( +)-glucose in aqueous solutions of [Ch][Sal] (0.0305 mol·kg^−1^)										
0.0000	1500.37	1512.32	1522.67	1531.46	1538.76	–	–	–	–	–
0.0252	1501.97	1513.88	1524.20	1532.90	1540.14	− 1.84	− 1.69	− 1.57	− 1.32	− 1.17
0.0503	1503.53	1515.38	1525.60	1534.30	1541.48	− 1.80	− 1.62	− 1.41	− 1.28	− 1.11
0.0750	1505.08	1516.84	1526.99	1535.64	1542.78	− 1.79	− 1.57	− 1.36	− 1.22	− 1.08
0.1003	1506.68	1518.39	1528.48	1537.00	1544.03	− 1.78	− 1.57	− 1.37	− 1.18	− 1.01
0.1247	1508.19	1519.81	1529.83	1538.35	1545.32	− 1.76	− 1.54	− 1.33	− 1.17	− 1.00
0.1500	1509.60	1521.31	1531.38	1539.62	1546.49	− 1.68	− 1.52	− 1.36	− 1.11	− 0.93
*D(* +*)*-glucose in aqueous solutions of [Ch][Sal] (0.0600 mol·kg^−1^*)*										
0.0000	1503.98	1515.71	1525.77	1534.30	1541.37	–	–	–	–	–
0.0250	1505.56	1517.22	1527.24	1535.71	1542.73	− 1.79	− 1.56	− 1.42	− 1.25	− 1.11
0.0500	1507.13	1518.72	1528.66	1537.11	1544.10	− 1.77	− 1.54	− 1.35	− 1.22	− 1.10
0.075	1508.67	1520.20	1530.10	1538.52	1545.41	− 1.73	− 1.51	− 1.34	− 1.22	− 1.04
0.0999	1510.22	1521.67	1531.52	1539.89	1546.71	− 1.72	− 1.48	− 1.31	− 1.18	− 1.01
0.1248	1511.77	1523.26	1533.17	1541.30	1548.03	− 1.71	− 1.52	− 1.40	− 1.18	− 1.00
0.1499	1513.29	1524.65	1534.34	1542.60	1549.21	− 1.68	− 1.46	− 1.27	− 1.13	− 0.92
*D(* +*)*-glucose in aqueous solutions of [Ch][Sal] (0.0900 mol·kg^−1^*)*										
0.0000	1507.38	1518.87	1528.80	1537.10	1544.20	–	–	–	–	–
0.0250	1509.00	1520.45	1530.32	1538.57	1545.61	− 1.84	− 1.69	− 1.50	− 1.35	− 1.19
0.0491	1510.57	1521.93	1531.75	1539.92	1546.92	− 1.84	− 1.62	− 1.45	− 1.26	− 1.11
0.0745	1512.14	1523.44	1533.19	1541.32	1548.23	− 1.77	− 1.55	− 1.37	− 1.20	− 1.03
0.0999	1513.72	1524.99	1534.60	1542.71	1549.56	− 1.73	− 1.54	− 1.31	− 1.16	− 0.99
0.1244	1515.22	1526.39	1535.98	1544.00	1550.81	− 1.69	− 1.48	− 1.28	− 1.12	− 0.95
0.1499	1516.80	1527.80	1537.33	1545.15	1551.73	− 1.67	− 1.42	− 1.23	− 1.01	− 0.78
*D(* +*)*-glucose in aqueous solutions of [Ch][For] (0.0297 mol·kg^−1^*)*										
0.0000	1498.99	1511.75	1523.20	1532.85	1541.75	–	–	–	–	–
0.0251	1500.72	1513.40	1524.77	1534.33	1543.13	− 2.33	− 2.04	− 1.77	− 1.51	− 1.23
0.0504	1502.41	1515.06	1526.38	1535.79	1544.49	− 2.24	− 2.01	− 1.77	− 1.44	− 1.17
0.0752	1504.12	1516.63	1527.74	1537.18	1545.78	− 2.22	− 1.91	− 1.58	− 1.36	− 1.10
0.1003	1505.56	1518.12	1529.22	1538.41	1547.01	− 2.02	− 1.81	− 1.53	− 1.22	− 1.01
0.1252	1507.13	1519.59	1530.52	1539.71	1548.21	− 1.97	− 1.73	− 1.42	− 1.15	− 0.94
0.1499	1508.74	1520.91	1531.76	1540.86	1549.16	− 1.93	− 1.61	− 1.31	− 1.06	− 0.79
*D(* +*)*-glucose in aqueous solutions of [Ch][For] (0.0600 mol·kg^−1^*)*										
0.0000	1501.24	1513.25	1523.97	1534.08	1543.78	–	–	–	–	–
0.0252	1502.82	1514.78	1525.46	1535.53	1545.19	− 1.83	− 1.65	− 1.49	− 1.34	− 1.21
0.0501	1504.39	1516.31	1526.93	1536.93	1546.53	− 1.83	− 1.65	− 1.47	− 1.30	− 1.14
0.0751	1505.95	1517.80	1528.40	1538.38	1547.89	− 1.80	− 1.61	− 1.45	− 1.30	− 1.11
0.1001	1507.46	1519.23	1529.79	1539.69	1549.21	− 1.75	− 1.54	− 1.38	− 1.21	− 1.06
0.1248	1508.92	1520.75	1531.18	1541.11	1550.55	− 1.69	− 1.54	− 1.35	− 1.20	− 1.04
0.1502	1510.56	1522.20	1532.68	1542.47	1551.81	− 1.70	− 1.49	− 1.34	− 1.17	− 0.99
*D(* +*)*-glucose in aqueous solutions of [Ch][For] (0.0900 mol·kg^−1^*)*										
0.0000	1503.28	1515.47	1526.11	1535.27	1542.86	–	–	–	–	–
0.0253	1504.91	1517.03	1527.60	1536.68	1544.21	− 1.88	− 1.65	− 1.43	− 1.20	− 1.02
0.0498	1506.45	1518.52	1529.05	1538.06	1545.49	− 1.82	− 1.62	− 1.43	− 1.20	− 0.98
0.0747	1508.07	1520.02	1530.46	1539.39	1546.79	− 1.83	− 1.58	− 1.37	− 1.15	− 0.96
0.0999	1509.67	1521.50	1531.91	1540.77	1548.06	− 1.83	− 1.55	− 1.36	− 1.14	− 0.93
0.1245	1511.14	1522.97	1533.22	1542.05	1549.32	− 1.77	− 1.53	− 1.29	− 1.10	− 0.91
0.1497	1512.73	1524.46	1534.74	1543.33	1550.58	− 1.75	− 1.51	− 1.31	− 1.05	− 0.89
*D(* +*)*-glucose in aqueous solutions of [Ch][Ace] (0.0299 mol·kg^−1^*)*										
0.0000	1500.33	1512.35	1522.80	1531.81	1538.97	–	–	–	–	–
0.0254	1501.93	1513.89	1524.24	1533.16	1540.20	− 1.82	− 1.62	− 1.34	− 1.11	− 0.83
0.0499	1503.46	1515.35	1525.59	1534.36	1541.35	− 1.79	− 1.58	− 1.29	− 0.99	− 0.77
0.0752	1505.02	1516.86	1527.01	1535.64	1542.56	− 1.76	− 1.56	− 1.28	− 0.96	− 0.77
0.1000	1506.55	1518.32	1528.41	1536.92	1543.74	− 1.73	− 1.53	− 1.28	− 0.96	− 0.75
0.1251	1508.08	1519.78	1529.82	1538.22	1544.94	− 1.71	− 1.50	− 1.27	− 0.96	− 0.75
0.1500	1509.58	1521.22	1531.21	1539.57	1546.08	− 1.68	− 1.48	− 1.26	− 0.98	− 0.72
*D(* +*)*-glucose in aqueous solutions of [Ch][Ace] (0.0600 mol·kg^−1^*)*										
0.0000	1503.59	1515.49	1525.71	1534.32	1541.58	–	–	–	–	–
0.0257	1505.21	1517.05	1527.19	1535.74	1542.93	− 1.80	− 1.61	− 1.38	− 1.21	− 1.03
0.0499	1506.73	1518.45	1528.60	1537.07	1544.16	− 1.78	− 1.51	− 1.39	− 1.19	− 0.97
0.0750	1508.28	1519.93	1529.97	1538.42	1545.46	− 1.75	− 1.49	− 1.31	− 1.15	− 0.97
0.0997	1509.81	1521.37	1531.38	1539.77	1546.76	− 1.72	− 1.47	− 1.31	− 1.14	− 0.97
0.1248	1511.32	1522.84	1532.81	1541.15	1548.07	− 1.69	− 1.45	− 1.30	− 1.14	− 0.96
0.1500	1512.85	1524.33	1534.17	1542.53	1549.40	− 1.66	− 1.45	− 1.26	− 1.13	− 0.96
*D(* +*)*-glucose in aqueous solutions of [Ch][Ace] (0.0900 mol·kg^−1^*)*										
0.0000	1507.04	1518.69	1528.76	1537.15	1544.16	–	–	–	–	–
0.0250	1508.56	1520.16	1530.18	1538.52	1545.46	− 1.63	− 1.45	− 1.29	− 1.14	− 0.96
0.0502	1510.08	1521.63	1531.60	1539.86	1546.77	− 1.61	− 1.43	− 1.27	− 1.09	− 0.94
0.0750	1511.58	1523.06	1532.99	1541.20	1548.06	− 1.60	− 1.40	− 1.25	− 1.08	− 0.94
0.1000	1513.10	1524.56	1534.41	1542.56	1549.37	− 1.59	− 1.42	− 1.25	− 1.08	− 0.94
0.1249	1514.61	1526.02	1535.78	1543.92	1550.66	− 1.58	− 1.41	− 1.22	− 1.08	− 0.93
0.1496	1516.12	1527.42	1537.18	1545.28	1551.97	− 1.58	− 1.38	− 1.22	− 1.08	− 0.93

^a^The standard uncertainties for molality, temperature and pressure were* u* (*m*) = 0.001 mol kg^−1^, *u* (*T*) = 0.2 K, *u* (*P*) = 10.5 hPa, respectively with level of confidence 0.95. The standard combined uncertainty for speed of sound and apparent molar compressibility were estimated to be, *u*_*c*_ (*u*) = 1.5 m s^−1^ and *u*_*c*_(*κ*_*φ*_) = 3.10^–13^ m^3^ mol·Pa^−1^ (level of confidence 0.68), respectively

**Fig. 4:**
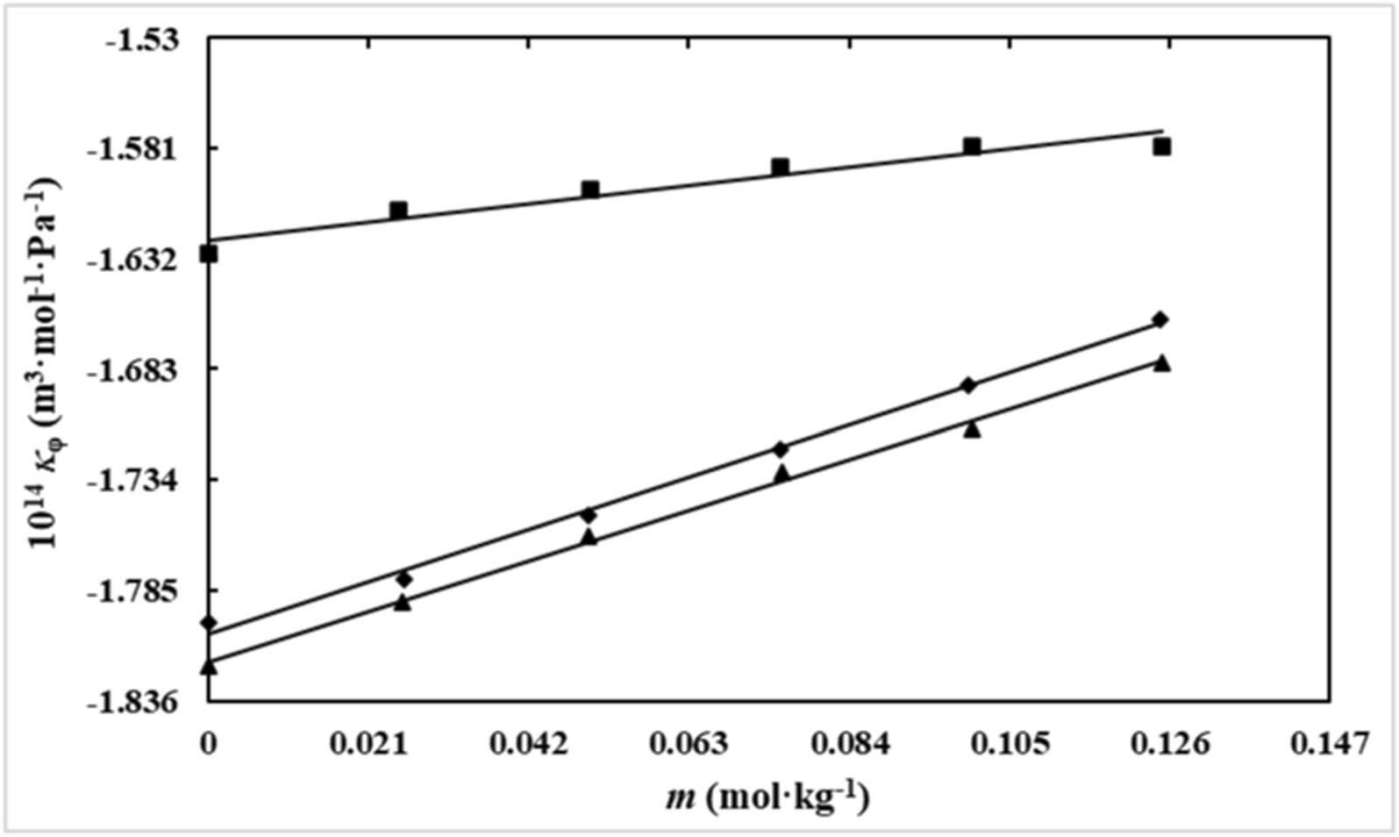
Apparent molar isentropic compressibility (*κ*_φ_), of *D(* +*)*-glucose versus its molality (*m* / mol.kg^−1^) in aqueous [Ch][Ace] solutions with varying molalities:∎, 0.0900; ◆, 0.0600; ▲, 0.0299 at *T* = 298.15 K

Generally, the speed of sound increased with rising IL and *D(* +*)*-glucose content, as well as with increasing temperature. The graphical representation of $\kappa_{\varphi }$ values for *D(* +*)*-glucose at different concentrations of aqueous [Ch][Ace] solutions has been depicted in Fig. 4.

The data reveal that $\kappa_{\varphi }$ values are negative at all studied temperatures and become more negative with increasing IL concentration. Literature reports that $\kappa_{\varphi }$ values in aqueous solutions typically exhibit (a) large negative values for ionic compounds, (b) positive values for primarily hydrophobic solutes, and (c) intermediate, small, and negative values for uncharged hydrophilic solutes like sugars [43, 44].

The dependence of $\kappa_{\varphi }$ on molal base concentration can be adequately represented by the following equation [63]:

9 $$\kappa_{\varphi } = \kappa_{\varphi }^{0} + S_{k} m$$

where $\kappa_{\varphi }^{0}$ is the limiting value of apparent molar isentropic compressibility, $S_{k}$ has its own empirical meanings similar to those in Eq. 2 for apparent molar volumes. The values of $\kappa_{\varphi }^{0}$,$S_{k}$, for the studied solutions, along with their standard deviations at the experimental temperatures, are presented in Table 7.

**Table 7:** The values of partial molar isentropic compressibility ($\kappa_{\varphi }^{0}$), experimental slope ($S_{k}$), partial molar isentropic compressibility of transfer ($\Delta_{tr} \kappa_{\varphi }^{0}$) for *D(* +*)*-glucose in the aqueous solutions of ionic liquids at different temperature^a^

*T* (K)	10^14^ *S*_k_ (m^3^·kg·mol^−2^·Pa^−1^)	10^14^ *κ*_φ_^0^ (m^3^·mol^−1^·Pa^−1^)	Δ*κ*_φ_^0^ (m^3^·mol^−1^·Pa^−1^)	*σ* (*κ*_φ_)
*D(* +*)*-glucose in water				
298.15	0.60 ± 0.036	− 1.74 ± 0.003	–	0.008
303.15	0.59 ± 0.056	− 1.68 ± 0.005	–	0.009
308.15	0.52 ± 0.071	− 1.63 ± 0.006	–	0.010
313.15	0.51 ± 0.049	− 1.59 ± 0.004	–	0.009
318.15	0.50 ± 0.061	− 1.56 ± 0.006	–	0.010
D( +)-glucose in aqueous solutions of [Ch][Sal] (0.0300 mol·kg^−1^)				
298.15	1.09 ± 0.215	− 1.87 ± 0.021	− 0.13	0.02
303.15	1.24 ± 0.224	− 1.69 ± 0.022	− 0.01	0.02
308.15	1.48 ± 0.564	− 1.53 ± 0.055	0.10	0.05
313.15	1.63 ± 0.114	− 1.35 ± 0.011	0.24	0.01
318.15	1.82 ± 0.122	− 1.21 ± 0.012	0.35	0.01
D( +)-glucose in aqueous solutions of [Ch][Sal] (0.0600 mol·kg^−1^)				
298.15	0.88 ± 0.077	− 1.81 ± 0.007	− 0.07	0.01
303.15	0.64 ± 0.207	− 1.57 ± 0.020	0.11	0.02
308.15	0.73 ± 0.474	− 1.42 ± 0.046	0.21	0.04
313.15	0.88 ± 0.121	− 1.28 ± 0.012	0.31	0.01
318.15	1.47 ± 0.167	− 1.16 ± 0.016	0.40	0.01
D( +)-glucose in aqueous solutions of [Ch][Sal] (0.0900 mol·kg^−1^)				
298.15	1.52 ± 0.170	− 1.89 ± 0.016	− 0.15	0.01
303.15	2.01 ± 0.139	− 1.73 ± 0.013	− 0.05	0.01
308.15	2.22 ± 0.130	− 1.55 ± 0.013	0.08	0.01
313.15	2.48 ± 0.197	− 1.40 ± 0.019	0.19	0.02
318.15	2.89 ± 0.351	− 1.26 ± 0.034	0.30	0.03
*D(* +*)*-glucose in aqueous solutions of [Ch][For] (0.0300 mol·kg^−1^)				
298.15	3.43 ± 0.387	− 2.42 ± 0.038	− 0.678	0.033
303.15	3.52 ± 0.243	− 2.16 ± 0.024	− 0.481	0.021
308.15	3.88 ± 0.365	− 1.90 ± 0.036	− 0.274	0.031
313.15	3.72 ± 0.192	− 1.62 ± 0.019	− 0.026	0.016
318.15	3.39 ± 0.257	− 1.34 ± 0.025	0.224	0.022
*D(* +*)*-glucose in aqueous solutions of [Ch][For] (0.0600 mol·kg^−1^)				
298.15	1.29 ± 0.190	− 1.88 ± 0.010	− 0.140	0.016
303.15	1.37 ± 0.206	− 1.70 ± 0.020	− 0.018	0.018
308.15	1.40 ± 0.156	− 1.53 ± 0.015	0.095	0.013
313.15	1.44 ± 0.180	− 1.38 ± 0.018	0.212	0.015
318.15	1.59 ± 0.127	− 1.23 ± 0.012	0.330	0.011
*D(* +*)*-glucose in aqueous solutions of [Ch][For] (0.0900 mol·kg^−1^)				
298.15	0.92 ± 0.203	− 1.89 ± 0.020	− 0.119	0.017
303.15	1.13 ± 0.058	− 1.67 ± 0.005	0.010	0.004
308.15	1.16 ± 0.229	− 1.47 ± 0.022	0.163	0.02
313.15	1.24 ± 0.167	− 1.25 ± 0.016	0.344	0.014
318.15	1.07 ± 0.079	− 1.04 ± 0.007	0.517	0.006
*D(* +*)*-glucose in aqueous solutions of [Ch][Ace] (0.0300 mol·kg^−1^)				
298.15	1.11 ± 0.026	− 1.83 ± 0.002	− 0.091	0.002
303.15	1.13 ± 0.050	− 1.59 ± 0.004	0.086	0.004
308.15	1.01 ± 0.157	− 1.41 ± 0.015	0.219	0.013
313.15	0.64 ± 0.455	− 1.22 ± 0.044	0.374	0.039
318.15	0.40 ± 0.126	− 1.01 ± 0120	0.548	0.011
*D(* +*)*-glucose in aqueous solutions of [Ch][Ace] (0.0600 mol·kg^−1^)				
298.15	1.36 ± 0.040	− 1.86 ± 0.030	− 0.123	0.011
303.15	1.13 ± 0.283	− 1.59 ± 0.028	0.086	0.024
308.15	1.01 ± 0.179	− 1.41 ± 0.017	0.219	0.015
313.15	0.64 ± 0.114	− 1.22 ± 0.011	0.374	0.009
318.15	0.40 ± 0.173	− 1.01 ± 0.017	0.548	0.015
*D(* +*)*-glucose in aqueous solutions of [Ch][Ace] (0.0900 mol·kg^−1^)				
298.15	0.35 ± 0.062	− 1.63 ± 0.006	0.112	0.005
303.15	0.44 ± 0.125	− 1.45 ± 0.012	0.227	0.011
308.15	0.57 ± 0.064	− 1.30 ± 0.006	0.330	0.005
313.15	0.40 ± 0.184	− 1.12 ± 0.018	0.465	0.016
318.15	0.24 ± 0.049	− 0.96 ± 0.004	0.601	0.004

^a^The standard uncertainties for molality, temperature and pressure were* u* (*m*) = 0.001 mol kg^−1^, *u* (*T*) = 0.2 K, *u* (*P*) = 10.5 hPa, respectively with level of confidence 0.95

The observed increase in $\kappa_{\varphi }^{0}$ values for *D*( +)-glucose with rising temperature and IL concentration is attributed to strong attractive interactions between *D*( +)-glucose and IL species. The transfer partial molar isentropic compressibility ($\Delta_{tr} \kappa_{\varphi }^{0}$) of *D*( +)-glucose from water to aqueous IL solutions at infinite dilution was calculated using the following formula [63]:

10 $$\Delta_{tr} \kappa_{\varphi }^{0} = \kappa_{\varphi }^{0} \,\left( {D(\, + ) - {\text{glucose in aqueous ILs}}} \right)\, - \kappa_{\varphi }^{0} \left( {D(\, + ) - {\text{glucose in water}}} \right)$$

The calculated $\Delta_{tr} \kappa_{\varphi }^{0}$ values, based on the co-sphere overlap model has been tabulated in Table 7. The positive values of $\Delta_{tr} \kappa_{\varphi }^{0}$ indicate the predominance of type (a) and (b) interactions. The generally negative and increasingly negative apparent molar compressibility values with rising IL content suggest that applying pressure induces a repulsive force in the bulk due to *D(* +*)*-glucose solvation. However, the system becomes more compressible with added IL. In conclusion, *D(* +*)*-glucose and Ionic liquids exhibit weak electrostatic interactions. These interactions are strengthened at elevated temperatures due to changes in bulk modulus, dehydration of ionic species, volume expansion, and a consequent decrease in water molecules surrounding the *D(* +*)*-glucose and IL ions, leading to intensified electrostatic interactions.

### Viscometric results

**Fig. 5:**
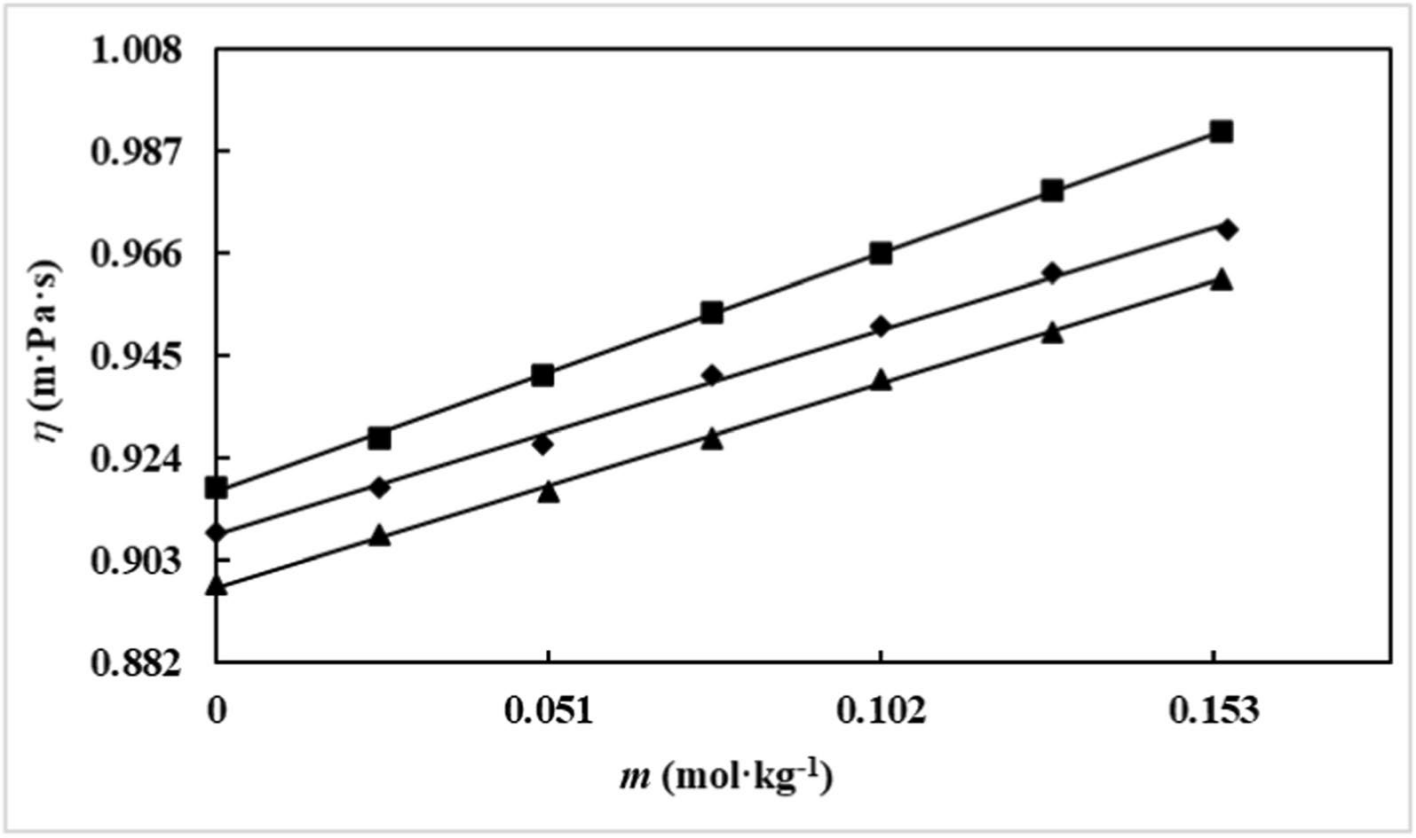
Viscosity of *D*( +)-glucose solutions as a function of molality (*m*/mol.kg^−1^) in aqueous [Ch][Ace] solutions with different molality concentrations of the ionic liquid: ∎ 0.0900; ◆ 0.0600; ▲ 0.0294 at* T* = 298.15 K

The experimental viscosity data (*η*) of aqueous solutions of *D(* +*)*-glucose in three ILs (0.05, 0.10, and 0.15 mol∙kg⁻^1^) at temperatures ranging from (298.15 to 318.15) K have been given in Table 8 and depicted in Fig. 5.

**Table 8:** The viscosity (*η*) data of aqueous *D(* +*)*-glucose in water and aqueous ILs solutions at (288.15 to 318.15) K^a^

*m* (mol·kg^−1^)		10^−3^*η* (m·Pa·s)			
*T (K)*	298.15	303.15	308.15	313.15	318.15
*D(* +*)*-glucose in aqueous solutions of [Ch][Sal] (0.0292 mol·kg^−1^*)*					
0.0000	0.884	0.800	0.717	0.665	0.605
0.0250	0.895	0.807	0.725	0.664	0.605
0.0510	0.908	0.817	0.736	0.669	0.610
0.0760	0.920	0.826	0.747	0.673	0.616
0.0960	0.930	0.833	0.756	0.676	0.620
0.1280	0.945	0.845	0.765	0.681	0.627
0.1540	0.958	0.856	0.770	0.692	0.632
*D(* +*)*-glucose in aqueous solutions of [Ch][Sal] (0.0597 mol·kg^−1^*)*					
0.0000	0.9094	0.855	0.745	0.673	0.615
0.0250	0.920	0.861	0.753	0.676	0.617
0.0510	0.930	0.868	0.76	0.683	0.623
0.0710	0.938	0.874	0.765	0.688	0.628
0.1010	0.949	0.882	0.773	0.695	0.635
0.1280	0.959	0.890	0.779	0.702	0.641
0.1540	0.964	0.897	0.786	0.708	0.647
*D(* +*)*-glucose in aqueous solutions of [Ch][Sal] (0.0899 mol·kg^−1^*)*					
0.0000	0.929	0.858	0.797	0.717	0.645
0.0250	0.940	0.864	0.802	0.721	0.650
0.0520	0.951	0.872	0.809	0.729	0.659
0.0760	0.961	0.878	0.816	0.736	0.667
0.1020	0.969	0.885	0.823	0.743	0.676
0.1280	0.980	0.892	0.830	0.751	0.685
0.1540	0.984	0.900	0.837	0.758	0.693
*D(* +*)*-glucose in aqueous solutions of [Ch][For] (0.0294 mol·kg^−1^*)*					
0.0000	0.894	0.811	0.725	0.635	0.545
0.028	0.902	0.816	0.730	0.644	0.558
0.051	0.912	0.823	0.738	0.655	0.569
0.076	0.922	0.831	0.747	0.667	0.581
0.102	0.932	0.838	0.755	0.679	0.592
0.128	0.942	0.846	0.764	0.692	0.604
0.153	0.952	0.853	0.772	0.704	0.616
*D(* +*)*-glucose in aqueous solutions of [Ch][For] (0.0605 mol·kg^−1^)					
0.0000	0.9033	0.811	0.7304	0.665	0.612
0.0250	0.911	0.817	0.735	0.669	0.614
0.0500	0.922	0.826	0.744	0.676	0.620
0.0760	0.932	0.834	0.752	0.684	0.626
0.1020	0.942	0.843	0.761	0.692	0.632
0.1280	0.952	0.851	0.770	0.699	0.639
0.1630	0.965	0.862	0.781	0.709	0.647
*D(* +*)*-glucose in aqueous solutions of [Ch][For] (0.0903 mol·kg^−1^)					
0.0000	0.910	0.821	0.748	0.681	0.624
0.0250	0.929	0.847	0.771	0.690	0.631
0.0510	0.941	0.862	0.761	0.696	0.634
0.0760	0.954	0.868	0.773	0.706	0.644
0.1020	0.966	0.883	0.794	0.723	0.659
0.1250	0.979	0.899	0.807	0.729	0.666
0.1550	0.991	0.914	0.815	0.735	0.679
*D(* +*)*-glucose in aqueous solutions of [Ch][Ace] (0.0294 mol·kg^−1^)					
0.0000	0.898	0.805	0.730	0.669	0.621
0.0250	0.908	0.815	0.736	0.672	0.621
0.0510	0.917	0.825	0.744	0.677	0.625
0.0760	0.928	0.837	0.753	0.685	0.630
0.1020	0.940	0.850	0.763	0.691	0.637
0.1280	0.950	0.860	0.772	0.696	0.641
0.1540	0.961	0.873	0.783	0.703	0.645
*D(* +*)*-glucose in aqueous solutions of [Ch][Ace] (0.0598 mol·kg^−1^)					
0.0000	0.909	0.820	0.727	0.676	0.621
0.0250	0.918	0.825	0.751	0.718	0.677
0.0500	0.927	0.836	0.768	0.722	0.686
0.0760	0.941	0.845	0.783	0.739	0.693
0.1020	0.951	0.854	0.794	0.743	0.701
0.1280	0.962	0.864	0.809	0.759	0.709
0.1550	0.971	0.871	0.820	0.765	0.715
*D(* +*)*-glucose in aqueous solutions of [Ch][Ace] (0.0896 mol·kg^−1^)					
0.0000	0.918	0.824	0.739	0.695	0.625
0.0250	0.928	0.837	0.770	0.698	0.631
0.0500	0.941	0.852	0.760	0.702	0.634
0.0760	0.954	0.857	0.772	0.709	0.644
0.1020	0.966	0.871	0.793	0.713	0.649
0.1280	0.979	0.889	0.806	0.719	0.656
0.1540	0.991	0.904	0.815	0.725	0.669

^a^The standard uncertainties for molality, temperature and pressure were* u* (*m*) = 0.001 mol kg^−1^, *u* (*T*) = 0.2 K, *u* (*P*) = 10.5 hPa, respectively with level of confidence 0.95. The standard combined uncertainty for viscosity was about, *u*_*c*_ (*η*) = 0.02 m.Pa.s (level of confidence 0.68)

As shown in Table 8 and Fig. 4, the viscosity of the ILs increases with increasing molecular weight but decreases with increasing temperature. Additionally, viscosity is observed to increase with an increment in both *D(* +*)*-glucose and IL concentration. The variation in relative viscosity (*η*_r_) of *D(* +*)*-glucose in water and aqueous Ionic liquids solutions can be described by the Jones–Dole equation [59]:

11 $$\frac{\eta }{{\eta_{0} }} = 1 + Ac^{1/2} + Bc$$

In the Jones-Dole equation, the Falkenhagen coefficient (*A*) and viscosity *B*-coefficient (*B*) are employed to characterize solute–solvent interactions. Additionally, the variable *c* represents the molar concentration of D(+)-glucose in an aqueous ionic liquid solution. While the viscosity *B*-coefficient is crucial for understanding these interactions, influenced by solute size, shape, and charge, the Falkenhagen coefficient, determined through least-squares fitting, was found to be negligible in our systems due to weak solute–solute interactions. Consequently, the Falkenhagen term was omitted, simplifying the equation to the following equation [59]:

12 $$\frac{\eta }{{\eta_{0} }} = 1 + Bc$$

where *η* and $\eta_{0}$ are the viscosities of the solution (*D(* +*)*-glucose in aqueous IL) and solvent (aqueous Ionic liquids), respectively, and c is the molar concentration of *D(* +*)*-glucose in the aqueous IL solution. Viscosity *B*-coefficients were determined from the slope of the linear plot of $(\eta /\eta_{0} - 1)$ vs. *c* using the least-squares method. The calculated viscosity *B*-coefficients and $\eta_{0}$ values for the studied solutions, obtained from fitting the experimental viscosity data to the Jones-Dole equation, are presented in Table 9.

**Table 9:** The viscosity *B*-coefficient values for aqueous solutions of *D(* +*)*-glucose in ILs at (288.15 – 318.15) K^a^

*T* (K)	*A*	*B* (dm^3/2^·mol^−1/2^)	*10*^*3*^*(dB/dT)*	*σ* (η)
*D(* +*)*-glucose in aqueous solutions of [Ch][Sal] (0.0294 mol·kg-1)				
298.15	− 0.013	0.585 ± 0.05	− 14.567	0.02
303.15	− 0.029	0.530 ± 0.02	− 10.654	0.02
308.15	0.013	0.481 ± 0.03	− 6.528	0.01
313.15	− 0.08	0.456 ± 0.08	− 2.188	0.01
318.15	− 0.061	0.462 ± 0.06	2.366	0.01
*D(* +*)*-glucose in aqueous solutions of [Ch][Sal] (0.0600 mol·kg-1)				
298.15	0.022	0.354 ± 0.01	2.496	0.02
303.15	− 0.013	0.363 ± 0.06	4.441	0.01
308.15	− 0.029	0.393 ± 0.04	6.474	0.01
313.15	− 0.036	0.441 ± 0.01	8.595	0.01
318.15	− 0.051	0.478 ± 0.01	10.803	0.03
*D(* +*)*-glucose in aqueous solutions of [Ch][Sal] (0.0900 mol·kg-1)				
298.15	0.031	0.324 ± 0.09	− 4.454	0.028
303.15	− 0.007	0.337 ± 0.01	4.500	0.021
308.15	− 0.015	0.372 ± 0.02	13.896	0.019
313.15	− 0.03	0.461 ± 0.03	23.735	0.005
318.15	− 0.045	0.615 ± 0.04	34.018	0.015
*D(* +*)*-glucose in aqueous solutions of [Ch][For] (0.0294 mol·kg^−1^)				
298.15	− 0.022	0.495 ± 0.05	− 2.484	0.025
303.15	− 0.03	0.432 ± 0.06	11.843	0.012
308.15	− 0.038	0.534 ± 0.07	26.873	0.034
313.15	− 0.053	0.866 ± 0.01	42.607	0.011
318.15	− 0.06	0.960 ± 0.06	59.046	0.018
*D(* +*)*-glucose in aqueous solutions of [Ch][For] (0.0600 mol·kg-1)				
298.15	− 0.012	0.458 ± 0.01	9.866	0.014
303.15	− 0.02	0.448 ± 0.03	6.438	0.008
308.15	− 0.035	0.522 ± 0.02	2.823	0.009
313.15	− 0.042	0.525 ± 0.05	− 0.980	0.005
318.15	− 0.05	0.487 ± 0.03	− 4.970	
*D(* +*)*-glucose in aqueous solutions of [Ch][For] (0.0900 mol·kg-1)				
298.15	0.054	0.450 ± 0.07	6.145	0.023
303.15	0.096	0.481 ± 0.05	11.890	0.015
308.15	− 0.013	0.629 ± 0.03	17.903	0.016
313.15	− 0.025	0.616 ± 0.03	24.185	0.013
318.15	− 0.098	0.834 ± 0.06	30.736	0.011
*D(* +*)*-glucose in aqueous solutions of [Ch][Ace] (0.0299 mol·kg-1)				
298.15	− 0.018	0.512 ± 0.01	14.371	0.021
303.15	− 0.018	0.607 ± 0.02	4.428	0.016
308.15	− 0.045	0.591 ± 0.03	− 6.029	0.012
313.15	− 0.048	0.464 ± 0.05	− 17.001	0.010
318.15	− 0.059	0.425 ± 0.04	− 28.487	0.0086
*D(* +*)*-glucose in aqueous solutions of [Ch][Ace] (0.0600 mol·kg-1)				
298.15	− 0.013	0.496 ± 0.06	40.090	0.025
303.15	− 0.021	0.481 ± 0.02	− 4.801	0.019
308.15	0.138	0.495 ± 0.03	− 51.963	0.015
313.15	0.332	0.009 ± 0.02	− 101.397	0.012
318.15	0.611	− 0.600 ± 0.07	− 153.103	
*D(* +*)*-glucose in aqueous solutions of [Ch][For] (0.0299 mol·kg-1)				
298.15	− 0.012	0.558 ± 0.06	− 19.797	0.004
303.15	− 0.019	0.670 ± 0.01	− 12.409	0.007
308.15	0.069	0.490 ± 0.03	− 4.629	0.080
313.15	− 0.038	0.382 ± 0.05	3.545	0.026
318.15	− 0.058	0.592 ± 0.06	12.111	0.046

^a^The standard uncertainties for molality, temperature and pressure were* u* (*m*) = 0.001 mol kg^−1^, *u* (*T*) = 0.2 K, *u* (*P*) = 10.5 hPa, respectively with level of confidence 0.95

The viscosity *B*-coefficient provides insights into solute size, shape, charge, and the structural effects induced by solute–solvent interactions [64–66]. The viscosity *B*-coefficient, a measure of solvation and its influence on solvent structure, reflects the net impact of charged end groups and hydrophilic/hydrophobic groups on solvent molecules. Positive viscosity *B*-coefficients for *D(* +*)*-glucose indicate a pronounced kosmotropic effect in aqueous choline based-ILs solutions, suggesting strong solute–solvent interactions within the studied systems. These strong interactions could potentially enhance the efficiency of choline-based ionic liquids in sugar conversion to bioethanol.

### Electrical conductivity results

**Fig. 6:**
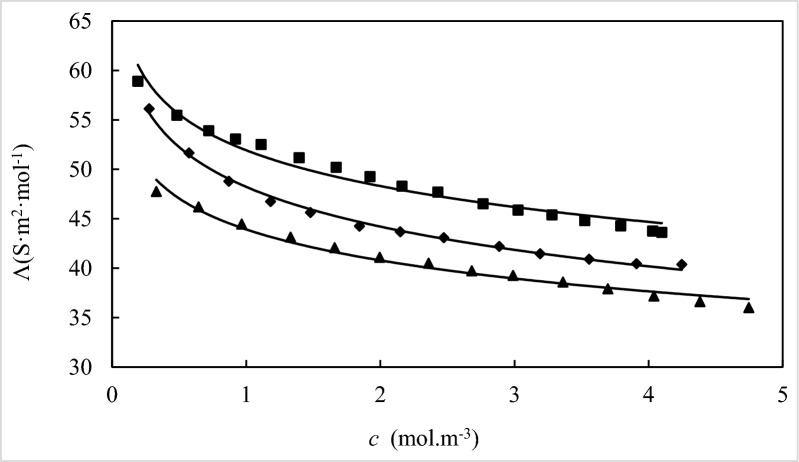
Molar conductivities (*Λ*) of [Ch][Ace] in aqueous *D(* +*)*-glucose solutions with different molality concentrations of *D(* +*)*-glucose:∎, 0.0500; ◆, 0.1000; ▲, 0.1500 at *T* = 298.15 K

The molar conductivity ($\Lambda$) values of choline-based ILs in varying concentrations of aqueous *D(* +*)*-glucose solutions. The graphical illustration of the dependence of $\Lambda$ on IL concentration at various *D(* +*)*-glucose molalities has been presented in Fig. 6.

As depicted in the Tables 10 and Fig. 5, the molar conductivity exhibits a clear decreasing trend as the concentrations of both *D(* +*)*-glucose and the choline based-ILs increase. To analyze the experimental data, the low concentration Chemical Model (lcCM) was employed using the following equations [67]:

13 $$\Lambda = \alpha \left[ {\Lambda_{0} - S\left( {c\alpha } \right)^{{{\raise0.7ex\hbox{$1$} \!\mathord{\left/ {\vphantom {1 2}}\right.\kern-0pt} \!\lower0.7ex\hbox{$2$}}}} + Ec\alpha \ln (c\alpha ) + J_{1} c\alpha + J_{2} (c\alpha )^{{{\raise0.7ex\hbox{$3$} \!\mathord{\left/ {\vphantom {3 2}}\right.\kern-0pt} \!\lower0.7ex\hbox{$2$}}}} } \right]$$

14 $$K_{A} = \frac{1 - \alpha }{{\alpha^{2} c\gamma_{ \pm }^{2} }}$$

15 $$\ln \gamma_{ \pm } = - \frac{kq}{{1 + kR}}$$

16 $$k^{2} = \frac{{16000N_{A} z^{2} e^{2} \alpha c}}{{\varepsilon_{0} \varepsilon k_{B} T}}$$

17 $$q = \frac{{z^{2} e^{2} }}{{8\pi \varepsilon_{0} \varepsilon k_{B} T}}$$

where $\Lambda$ is the molar conductivity, $\Lambda_{0}$ is the limiting molar conductivity, ($1 - \alpha$) is the fraction of oppositely charged ions acting as ion pairs, *R* is a distance parameter, and $\gamma_{ \pm }$ is the corresponding mean activity coefficient of free ions. The necessary parameters for calculating ($1 - \alpha$), $\gamma_{ \pm }$, and *R* were obtained from reference [67]. In this equation, *c* is the molar concentration of *D(* +*)*-glucose calculated from solution molality and density values. The remaining parameters hold their standard meanings. Nonlinear least-squares iteration applied to the molar conductivity data yielded the ion-association constant (*K*_A_), $\Lambda_{0}$, and *R*, as summarized in Table 11.

**Table 10:** The specific conductivity (*κ*), molar conductivity ($\Lambda$), of choline-based ILs in various concentrations of aqueous *D(* +*)*-glucose solutions at 298.15 K^a^

*m*_glucose_ (mol·kg^−1^)											
0.0000			0.0499			0.1003			0.1498		
*c*	*κ*	*Λ*	*c*	*κ*	*Λ*	*c*	*κ*	*Λ*	*c*	*κ*	*Λ*
(mol.m^−3^)	(μS.cm^−1^)	(S.cm^2^.mol^−1^)	(mol.m^−3^)	(μS.cm^−1^)	(S.cm^2^.mol^−1^)	(mol·m^−3^)	(μS.cm^−1^)	(S.cm^2^.mol^−1^)	(mol·m^−3^)	(μS.cm^−1^)	(S.cm^2^.mol^−1^)
[Ch][Sal]											
0.5557	37.49	67.458	0.4	25.5	63.751	0.6479	40.35	62.285	0.7079	43.69	61.712
0.7441	49.68	66.769	0.561	35.52	63.324	0.8217	50.79	61.81	0.9027	54.98	60.902
0.9186	60.71	66.09	0.7817	49.03	62.727	1.0142	62.15	61.279	1.1308	68.08	60.204
1.0748	70.6	65.689	0.961	59.84	62.271	1.1926	72.15	60.496	1.3493	80.18	59.42
1.2585	81.81	65.007	1.0898	67.58	62.007	1.432	85.19	59.493	1.5299	90.17	58.937
1.456	93.82	64.438	1.2415	76.49	61.613	1.695	99.81	58.884	1.7722	103.59	58.451
1.5846	101.79	64.238	1.3978	85.5	61.168	1.8734	109.61	58.507	2.0383	117.59	57.69
1.7637	111.99	63.497	1.6048	97.41	60.697	2.1082	121.81	57.779	2.2188	126.99	57.232
1.9245	121.49	63.13	1.7887	108	60.381	2.3664	135.61	57.305	2.4469	138.69	56.679
1.9933	125.69	63.055	2.014	120.8	59.982	2.6199	148.61	56.722	2.6512	148.89	56.159
2.2689	141.29	62.272	2.2301	132.9	59.595	2.8312	159.51	56.339	2.8602	158.19	55.306
2.4664	151.79	61.543	2.4232	143.5	59.22						
2.641	162.19	61.413	2.6485	155.8	58.826						
[Ch][For]											
0.3039	22.32	73.439	0.2548	18.13	71.158	0.4164	28.36	68.111	0.3303	21.43	64.884
0.673	48.55	72.143	0.6968	48.84	70.095	0.7722	51.6	66.823	0.7528	48.19	64.015
1.0131	72.21	71.274	1.1089	76.54	69.021	1.1886	78.39	65.953	1.1523	71.79	62.303
1.2302	86.93	70.666	1.491	100.94	67.7	1.605	104.21	64.929	1.4826	91.31	61.588
1.5848	110.8	69.917	1.8657	124.35	66.65	2.0289	130.18	64.163	1.9666	118.83	60.425
1.8816	130.41	69.308	2.2628	148.65	65.695	2.5513	160.93	63.076	2.4583	146.49	59.589
2.3447	160.51	68.455									
[Ch][Ace]											
0.2574	14.93	58.006	0.1915	11.28	58.912	0.2763	15.51	56.132	0.3291	15.71	47.751
0.4591	26.37	57.449	0.4826	26.77	55.472	0.5732	29.61	51.666	0.6441	29.77	46.212
0.6887	38.56	55.99	0.7201	38.82	53.911	0.8702	42.47	48.8	0.9662	42.96	44.463
0.9113	49.71	54.548	0.9193	48.78	53.064	1.181	55.21	46.745	1.3303	57.39	43.142
1.1896	62.99	52.951	1.1108	58.33	52.513	1.478	67.45	45.634	1.6593	69.81	42.071
1.5444	79.02	51.166	1.3943	71.34	51.168	1.844	81.62	44.26	1.9954	82.03	41.11
1.7809	89.64	50.334	1.6701	83.85	50.209	2.1479	93.82	43.678	2.3595	95.59	40.513
2.0453	101.04	49.402	1.9229	94.73	49.266	2.4725	106.52	43.08	2.6815	106.55	39.736
2.3862	115.64	48.463	2.1604	104.33	48.294	2.8869	121.82	42.196	2.9896	117.4	39.268
2.6853	127.54	47.496	2.4285	115.83	47.697	3.1908	132.32	41.468	3.3607	129.75	38.607
3.047	141.84	46.55	2.7656	128.63	46.512	3.5569	145.52	40.912	3.6967	140.14	37.91
3.4714	157.84	45.469	3.0261	138.83	45.879	3.9091	158.12	40.449	4.0398	150.25	37.193
3.9375	174.94	44.429	3.2789	148.83	45.391	4.2475	171.52	40.381	4.3829	160.51	36.621
			3.524	157.93	44.816				4.7469	170.92	36.006
			3.7922	167.93	44.284						
			4.0297	176.34	43.759						
			4.0986	178.83	43.633						

^a^The standard uncertainties for molality and temperature were* u* (*C*) = 0.001 mol m^−3^ and *u* (*T*) = 0.5 K, respectively with level of confidence 0.95. The standard combined uncertainty for conductance and molar conductivity were about, *u*_*c*_ (*κ*) = 0.5 μS.cm^−1^ and *u*_*c*_(*Λ*) = 0.7 μS.cm^2^.mol^−1^ (level of confidence 0.68), respectively

**Table 11:** The ion association constants (*K*_*A*_), limiting molar conductivities ($\Lambda_{0}$), the distance of closest approach of ions (*R*), and standard deviations (*S*_dev_ (*Λ*)) of IL in aqueous *D(* +*)*-glucose solutions at 298.15 K

*m* (mol·kg^−1^)	*K*_A_ (dm^3^·mol^−1^)	*Λ*_0_ (S.cm^2^.mol^−1^)	10^10^*R* (m)	*S*_*dev*_* (Λ)*
[Ch][Sal] in aqueous *D(* +*)*-glucose				
0.0000	41.300	70.135	34.11	_0.11_
0.0500	46.443	65.195	22.05	_0.05_
0.1000	65.742	65.148	10.40	_0.14_
0.1500	68.240	64.758	4.54	_0.15_
[Ch][For] in aqueous *D(* +*)*-glucose				
0.0000	37.073	74.616	26.47	_0.07_
0.0500	44.534	72.127	29.44	_0.27_
0.1000	45.837	69.557	2.787	_0.12_
0.1500	51.281	66.293	24.41	_0.27_
[Ch][Ace] in aqueous *D(* +*)*-glucose				
0.0000	124.082	60.58	29.66	_0.26_
0.0500	132.421	59.884	1.337	_0.38_
0.1000	165.962	56.285	19.34	_0.80_
0.1500	140.357	49.773	0.390	_0.20_

The estimated uncertainities for u (*K*_*A*_) = 0.3 dm^3^·mol, u (10^4^*Λ*_*0*_) = 0.075 S·m^2^·mol. (level of confidence 0.68)

The observed decrease in $\Lambda_{0}$ and increase in *K*_A_ with rising IL concentration can be attributed to two primary factors: (i) strengthened interactions between *D(* +*)*-glucose and choline based-IL ions, leading to larger solvated ion radii and reduced mobility, and (ii) increased solution viscosity due to higher IL content, hindering ion mobility [68, 69]. The enhanced electrostatic interactions between Ionic liquids and *D(* +*)*-glucose, arising from the increased number of IL ions, contribute to ion association. Moreover, the elevated solution viscosity reduces ionic mobility and diffusion, further promoting ion-pair formation in the studied systems.

### Taste behavioral results

The taste behavior of *D(* +*)*-glucose in the presence of water and aqueous ionic liquids solutions, the apparent specific volumes (*ASV*) and apparent specific isentropic compressibility (*ASIC*) of *D(* +*)*-glucose in varying aqueous ILs solutions has been investigated through the following expression [70]:

18 $$ASV = \frac{{V_{\varphi } }}{M}$$

19 $$ASIC = \frac{{\kappa_{\varphi } }}{M}$$

*M* is the molar mass of *D(* +*)*-glucose. The *ASV* and *ASIC* values of *D(* +*)*-glucose in both pure water and aqueous ionic liquid solutions (Table 12) suggest that the addition of the studied choline based-Ionic liquids does not significantly alter the physical properties related to the taste behavior of *D(* +*)*-glucose [70].

**Table 12:** The values of apparent specific volume (*ASV*) and apparent specific isentropic compressibility (*ASIC*)*,* values for *D(* +*)*-glucose in water and aqueous ionic liquids solutions at *T* = (288.15 to 318.15) K

*m* (mol·kg^−1^)	*ASV* (cm^3^·g^−1^)					10^14^ *ASIC* (m^3^·g^−1^·Pa^−1^)				
*T* (K)	298.15	303.15	308.15	313.15	318.15	298.15	303.15	308.15	313.15	318.15
*D(* +*)*-glucose in water										
0.0000	–	–	–	–	–	–	–	–	–	–
0.0250	0.6110	0.6127	0.6143	0.6159	0.6171	− 0.00960	− 0.00924	− 0.00895	− 0.00873	− 0.00860
0.0500	0.6135	0.6147	0.6162	0.6177	0.6191	− 0.00947	− 0.00916	− 0.00888	− 0.00866	− 0.00851
0.0741	0.6155	0.6163	0.6176	0.6195	0.6211	− 0.00940	− 0.00911	− 0.00886	− 0.00860	− 0.00844
0.1001	0.6170	0.6186	0.6198	0.6214	0.6230	− 0.00930	− 0.00895	− 0.00870	− 0.00848	− 0.00831
0.1249	0.6191	0.6208	0.6222	0.6236	0.6245	− 0.00925	− 0.00889	− 0.00862	− 0.00842	− 0.00831
0.1498	0.6212	0.6223	0.6234	0.6250	0.6262	− 0.00918	− 0.00886	− 0.00863	− 0.00840	− 0.00827
*D(* +*)*-glucose in aqueous solutions of [Ch][Sal] (0.0299 mol·kg^−1^*)*										
0.0000	–	–	–	–	–	–	–	–	–	–
0.0252	0.6206	0.6221	0.6240	0.6260	0.6272	− 0.01024	− 0.00938	− 0.00869	− 0.00733	− 0.00647
0.0503	0.6208	0.6224	0.6246	0.6261	0.6280	− 0.00997	− 0.00898	− 0.00784	− 0.00709	− 0.00617
0.0750	0.6207	0.6231	0.6254	0.6269	0.6280	− 0.00994	− 0.00871	− 0.00753	− 0.00676	− 0.00599
0.1003	0.6213	0.6234	0.6251	0.6269	0.6283	− 0.00988	− 0.00871	− 0.00762	− 0.00657	− 0.00561
0.1247	0.6215	0.6234	0.6259	0.6277	0.6289	− 0.00976	− 0.00853	− 0.00736	− 0.00647	− 0.00555
0.1500	0.6216	0.6235	0.6260	0.6280	0.6292	− 0.00933	− 0.00845	− 0.00753	− 0.00616	− 0.00516
*D(* +*)*-glucose in aqueous solutions of [Ch][Sal] (0.0600 mol·kg^−1^*)*										
0.0000	–	–	–	–	–	–	–	–	–	–
0.0250	0.6209	0.6228	0.6244	0.6262	0.6281	− 0.00992	− 0.00866	− 0.00790	− 0.00694	− 0.00617
0.0500	0.6210	0.6233	0.6249	0.6269	0.6294	− 0.00983	− 0.00854	− 0.00752	− 0.00680	− 0.00609
0.075	0.6213	0.6234	0.6252	0.6273	0.6298	− 0.00960	− 0.00838	− 0.00744	− 0.00675	− 0.00580
0.0999	0.6214	0.6240	0.6259	0.6279	0.6299	− 0.00953	− 0.00823	− 0.00729	− 0.00657	− 0.00563
0.1248	0.6216	0.6241	0.6261	0.6281	0.6303	− 0.00947	− 0.00845	− 0.00779	− 0.00657	− 0.00555
0.1499	0.6218	0.6242	0.6263	0.6286	0.6313	− 0.00930	− 0.00813	− 0.00706	− 0.00627	− 0.00511
*D(* +*)*-glucose in aqueous solutions of [Ch][Sal] (0.0900 mol·kg^−1^*)*										
0.0000	–	–	–	–	–	–	–	–	–	–
0.0250	0.6213	0.6231	0.6249	0.6269	0.6289	− 0.01021	− 0.00936	− 0.00833	− 0.00748	− 0.00658
0.0491	0.6216	0.6235	0.6253	0.6273	0.6294	− 0.01023	− 0.00902	− 0.00805	− 0.00700	− 0.00618
0.0745	0.6219	0.6238	0.6257	0.6278	0.6300	− 0.00980	− 0.00863	− 0.00760	− 0.00668	− 0.00571
0.0999	0.6222	0.6242	0.6261	0.6282	0.6305	− 0.00960	− 0.00855	− 0.00728	− 0.00647	− 0.00552
0.1244	0.6226	0.6246	0.6265	0.6286	0.6310	− 0.00939	− 0.00823	− 0.00710	− 0.00620	− 0.00529
0.1499	0.6228	0.6249	0.6269	0.6289	0.6315	− 0.00928	− 0.00789	− 0.00681	− 0.00560	− 0.00435
*D(* +*)*-glucose in aqueous solutions of [Ch][For] (0.0303 mol·kg^−1^*)*										
0.0000	–	–	–	–	–	–	–	–	–	–
0.0251	0.6120	0.6152	0.6180	0.6205	0.6231	-0.01293	− 0.01132	− 0.00982	− 0.00838	− 0.00683
0.0504	0.6133	0.6162	0.6198	0.6219	0.6238	-0.01243	− 0.01116	− 0.00982	− 0.00799	− 0.00649
0.0752	0.6152	0.6189	0.6212	0.6237	0.6256	-0.01232	− 0.01060	− 0.00877	− 0.00755	− 0.00611
0.1003	0.6168	0.6202	0.6228	0.6249	0.6269	-0.01121	− 0.01005	− 0.00849	− 0.00677	− 0.00561
0.1252	0.6178	0.6214	0.6240	0.6266	0.6283	-0.01093	− 0.00960	− 0.00788	− 0.00638	− 0.00522
0.1499	0.6202	0.6228	0.6254	0.6276	0.6295	-0.01071	− 0.00894	− 0.00727	− 0.00588	− 0.00438
*D(* +*)*-glucose in aqueous solutions of [Ch][For] (0.0600 mol·kg^−1^*)*										
0.0000	–	–	–	–	–	–	–	–	–	–
0.0252	0.6186	0.6207	0.6228	0.6249	0.6267	− 0.01016	− 0.00916	− 0.00827	− 0.00744	− 0.00672
0.0501	0.6190	0.6211	0.6236	0.6252	0.6271	− 0.01016	− 0.00916	− 0.00816	− 0.00722	− 0.00633
0.0751	0.6201	0.6218	0.6241	0.6262	0.6281	− 0.00999	− 0.00894	− 0.00805	− 0.00722	− 0.00616
0.1001	0.6207	0.6228	0.6250	0.6271	0.6289	− 0.00971	− 0.00855	− 0.00766	− 0.00672	− 0.00588
0.1248	0.6213	0.6234	0.6255	0.6279	0.6296	− 0.00938	− 0.00855	− 0.00749	− 0.00666	− 0.00577
0.1502	0.6222	0.6243	0.6266	0.6284	0.6299	− 0.00944	− 0.00827	− 0.00744	− 0.00649	− 0.00550
*D(* +*)*-glucose in aqueous solutions of [Ch][For] (0.0900 mol·kg^−1^*)*										
0.0000	–	–	–	–	–	–	–	–	–	–
0.0253	0.6213	0.6232	0.6251	0.6274	0.6292	− 0.01044	− 0.00916	− 0.00794	− 0.00666	− 0.00566
0.0498	0.6213	0.6231	0.6250	0.6274	0.6294	− 0.01010	− 0.00899	− 0.00794	− 0.00666	− 0.00544
0.0747	0.6217	0.6235	0.6255	0.6274	0.6295	− 0.01016	− 0.00877	− 0.00760	− 0.00638	− 0.00533
0.0999	0.6211	0.6231	0.6252	0.6277	0.6299	− 0.01016	− 0.00860	− 0.00755	− 0.00633	− 0.00516
0.1245	0.6216	0.6237	0.6258	0.6275	0.6298	− 0.00982	− 0.00849	− 0.00716	− 0.00611	− 0.00505
0.1497	0.6221	0.6239	0.6259	0.6283	0.6297	− 0.00971	− 0.00838	− 0.00727	− 0.00583	− 0.00494
*D(* +*)*-glucose in aqueous solutions of [Ch][Ace] (0.0305 mol·kg^−1^*)*										
0.0000	–	–	–	–	–	–	–	–	–	–
0.0254	0.6214	0.6229	0.6241	0.6250	0.6257	− 0.01010	− 0.00899	− 0.00744	− 0.00616	− 0.00461
0.0499	0.6217	0.6232	0.6244	0.6253	0.6261	− 0.00994	− 0.00877	− 0.00716	− 0.00550	− 0.00427
0.0752	0.6221	0.6234	0.6247	0.6257	0.6264	− 0.00977	− 0.00866	− 0.00710	− 0.00533	− 0.00427
0.1000	0.6223	0.6237	0.6250	0.6259	0.6267	− 0.00960	− 0.00849	− 0.00710	− 0.00533	− 0.00416
0.1251	0.6227	0.6239	0.6253	0.6263	0.6270	− 0.00949	− 0.00833	− 0.00705	− 0.00533	− 0.00416
0.1500	0.6229	0.6243	0.6255	0.6267	0.6274	− 0.00933	− 0.00821	− 0.00699	− 0.00544	− 0.00400
*D(* +*)*-glucose in aqueous solutions of [Ch][Ace] (0.0600 mol·kg^−1^*)*										
0.0000	–	–	–	–	–	–	–	–	–	–
0.0257	0.6216	0.6228	0.6242	0.6253	0.6267	− 0.00999	− 0.00894	− 0.00766	− 0.00672	− 0.00572
0.0499	0.6218	0.6234	0.6244	0.6259	0.6269	− 0.00988	− 0.00838	− 0.00772	− 0.00661	− 0.00538
0.0750	0.6222	0.6238	0.6247	0.6262	0.6271	− 0.00971	− 0.00827	− 0.00727	− 0.00638	− 0.00538
0.0997	0.6225	0.6240	0.6251	0.6265	0.6273	− 0.00955	− 0.00816	− 0.00727	− 0.00633	− 0.00538
0.1248	0.6227	0.6242	0.6253	0.6267	0.6275	− 0.00938	− 0.00805	− 0.00722	− 0.00633	− 0.00533
0.1500	0.6230	0.6244	0.6256	0.6268	0.6278	− 0.00921	− 0.00805	− 0.00699	− 0.00627	− 0.00533
*D(* +*)*-glucose in aqueous solutions of [Ch][Ace] (0.0900 mol·kg^−1^*)*										
0.0000	–	–	–	–	–	–	–	–	–	–
0.0250	0.6226	0.6243	0.6257	0.6272	0.6283	− 0.00905	− 0.00805	− 0.00716	− 0.00633	− 0.00533
0.0502	0.6226	0.6246	0.6259	0.6273	0.6291	− 0.00894	− 0.00794	− 0.00705	− 0.00605	− 0.00522
0.0750	0.6228	0.6247	0.6261	0.6274	0.6290	− 0.00888	− 0.00777	− 0.00694	− 0.00599	− 0.00522
0.1000	0.6229	0.6249	0.6263	0.6276	0.6292	− 0.00883	− 0.00788	− 0.00694	− 0.00599	− 0.00522
0.1249	0.6230	0.6251	0.6264	0.6278	0.6292	− 0.00877	− 0.00783	− 0.00677	− 0.00599	− 0.00516
0.1496	0.6232	0.6252	0.6266	0.6278	0.6296	− 0.00877	− 0.00766	− 0.00677	− 0.00599	− 0.00516

### Hydration number results

The hydration number values (through utilization of Eq. 21) of *D(* +*)*-glucose in both pure water and aqueous Ionic liquids solutions has been tabulated within Table 13.

**Table 13:** Hydration numbers ($n_{H}$), of *D(* +*)*-glucose in water and in various aqueous choline based Ionic liquids solutions at temperatures, *T* = (293.15–318.15) K

*m* (mol·kg^−1^)	*T*(K)				
	298.15	303.15	308.15	313.15	318.15
	$$n_{H}$$				
*D(* +*)*-glucose in water					
0.0000	5.776	5.211	4.628	4.186	3.863
*D(* +*)*-glucose in aqueous solutions of [Ch][Sal]					
0.0300	5.176	4.651	4.113	3.713	3.415
0.0600	5.152	4.615	4.100	3.694	3.379
0.0900	5.134	4.607	4.078	3.667	3.357
*D(* +*)*-glucose in aqueous solutions of [Ch][For]					
0.0300	5.724	5.066	4.432	3.975	3.626
0.0600	5.313	4.758	4.193	3.775	3.454
0.0900	5.127	4.598	4.067	3.643	3.327
*D(* +*)*-glucose in aqueous solutions of [Ch][Ace]					
0.0300	5.119	4.616	4.112	3.747	3.473
0.0600	5.088	4.612	4.11	3.720	3.435
0.0900	5.018	4.537	4.033	3.647	3.359

while the change in volume attributed to electrostriction is related to the number of water molecules associated with *D(* +*)*-glucose, termed the hydration number (*n*_*H*_), accurately quantifying the number of water molecules interacting with solute species remains challenging despite extensive structural and computational investigations. This study determined hydration numbers using the following equation [71]:

20 $$n_{H} = \frac{{V_{\phi }^{0} \left( {elect.} \right)}}{{V_{E}^{0} - V_{B}^{0} }}$$

where $V_{\phi }^{0} \left( {elect.} \right)$ represents the electrostriction partial molar volume resulting from *D(* +*)*-glucose hydration. $V_{\phi }^{0} \left( {elect.} \right)$ can be approximated using the $V_{\phi }^{0}$ of *D(* +*)*-glucose and its corresponding intrinsic partial molar volume, $V_{\phi }^{0}$(int.), according to the following formula [72]:

21 $$V_{\phi }^{0} \left( {elect.} \right) = V_{\phi }^{0} - V_{\phi }^{0} ({\text{int}} .)$$

where

22 $$V_{\phi }^{0} ({\text{int}} .) = \left( {\frac{0.7}{{0.634}}} \right).V_{\phi }^{0} (cryst.)$$

23 $$V_{\phi }^{0} (cryst.) = \left( {{\raise0.7ex\hbox{$M$} \!\mathord{\left/ {\vphantom {M {d_{cryst.} }}}\right.\kern-0pt} \!\lower0.7ex\hbox{${d_{cryst.} }$}}} \right)$$

in which $V_{\phi }^{0} (cryst.)$ represents the crystal molar volume of *D(* +*)*-glucose and *M* is its molar mass, 0.7 is the packing density for molecules in organic crystals, and 0.634 is the packing density for random packed spheres. The crystal density ($d_{cryst.}$) of *D(* +*)*-glucose is 1.544 g.cm^−3^.

The electrostriction partial molar volume ($V_{E}^{0} - V_{B}^{0}$) is a crucial parameter in estimating the hydration number. Its values at 298.15, 303.15, 308.15, 313.15, and 318.15 K were reported as − 3.3, − 3.61, − 4, − 4.35, and − 4.65 cm^3^.mol^−1^, respectively [71–73]. For 303.15, 313.15, and 318.15 K, ($V_{E}^{0} - V_{B}^{0}$) values were determined through linear regression. Here, $V_{E}^{0}$ water represents the molar volume of electrostricted water, and $V_{B}^{0}$ denotes the molar volume of bulk water. By applying these values to Eq. (21), the hydration numbers for *D(* +*)*-glucose were calculated at various temperatures. As indicated in Table 13, a clear trend of decreasing hydration number with increasing temperature emerges, suggesting an enhanced dehydration effect of the ionic liquid at elevated temperatures.

## Conclusions

Investigation of the interactions between *D(* +*)*-glucose and three choline based-ILs ([Ch][Sal], [Ch][For], and [Ch][Ace]) in aqueous media was conducted through volumetric, compressibility, viscosity, and electrical conductivity measurements. Apparent molar volume ($V_{\varphi }$) and apparent molar isentropic compressibility ($\kappa_{\varphi }$) values of *D(* +*)*-glucose in aqueous IL solutions, calculated from density and speed of sound data, were used to determine standard and transfer partial molar properties. Results indicated that interactions between *D(* +*)*-glucose and Ionic liquids intensified with increasing IL concentration. Derived transfer properties, $\Delta_{tr} V_{\varphi }^{0}$ and $\Delta_{tr} \kappa_{\varphi }^{0}$, suggested the predominance of hydrophilic-ionic and hydrophilic-hydrophilic interactions between *D(* +*)*-glucose and IL ions. Viscosity measurements revealed that the BB-coefficient of viscosity increased with higher concentrations of ionic liquids (ILs), following the order: [Ch][Sal] > [Ch][For] > [Ch][Ace]. This indicates that D( +)-glucose demonstrates the most favorable interactions with [Ch][Sal] in aqueous solutions. Conductometric studies demonstrated that increasing IL concentration led to a decrease in limiting molar conductivity $\Lambda_{0}$ and an increase in the ion association constant ($K_{A}$) for *D(* +*)*-glucose in aqueous IL solutions. This behavior was attributed to enhanced electrostatic interactions and increased solution viscosity at higher IL concentrations, promoting ion-pair formation. The calculations of ASV and ASIC indicate that the studiedionic liquids does not significantly affect the physical properties of *D(* +*)*-glucose, suggesting they may be suitable as potential additives for fastening the bioethanol production. In the present study it was revealed that the alkyl chain length of choline-based choline based-ILs significantly influences their physicochemical properties. Increasing chain length correlates with enhanced solvent interaction and altered electronic structure, as evidenced by trends in dielectric solvation energy, HOMO, and LUMO energies. The hydration number of *D(* +*)*-glucose, a measure of the water molecules associated with a glucose molecule in solution, is significantly influenced by both temperature and the concentration of aqueous choline based-ILs. As temperature increases, the kinetic energy of water molecules rises, leading to a weakening of the hydrogen bonds between water and *D(* +*)*-glucose. Consequently, fewer water molecules are bound to the *D(* +*)*-glucose molecule, resulting in a decrease in the hydration number. Furthermore, the presence of ionic liquids in the solution can disrupt the water structure around the *D(* +*)*-glucose molecule. The ions in the IL compete with *D(* +*)*-glucose for water molecules, reducing the number available for hydration. As the concentration of the ionic liquids increases, this competitive effect becomes more pronounced, leading to a further decrease in the hydration number of *D(* +*)*-glucose. In essence, elevated temperature and increased IL concentration contribute to a reduction in the number of water molecules associated with *D(* +*)*-glucose in solution.

## Supplementary Information

Additional file 1.

## Data Availability

The authors confirm that the data supporting the findings of this study are available within the manuscript, figures, tables and supporting information files.
