# Supplementary material for: Unraveling the effect of choline-based choline based ionic liquids on the physicochemical properties and taste behavior of D( +)-glucose in aqueous solutions
Source: BMC Chem. 2025 Feb 22;19(1):49. doi: 10.1186/s13065-025-01407-3 (PMC11847403; doi:10.1186/s13065-025-01407-3)
Supplement: Supplementary file 1 — Additional file 1. [file 13065_2025_1407_MOESM1_ESM.docx]

**Supporting Materials**

**Unraveling the effect of choline-based ionic liquids on taste behavior and physicochemical properties of D(+)-glucose**

**Sara Dorosti, Mohammad Bagheri, Hemayat Shekaari**^[[1]](#footnote-1)^**, Fariba Ghaffari, Masumeh Mokhtarpour**

***Department of Physical Chemistry, University of Tabriz, Tabriz, Iran***


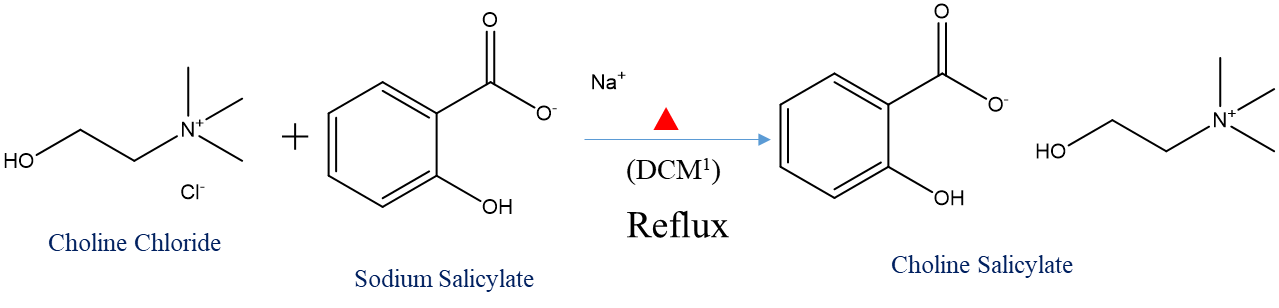


Fig S1. The synthesis plan of the [Ch][Sal] IL.


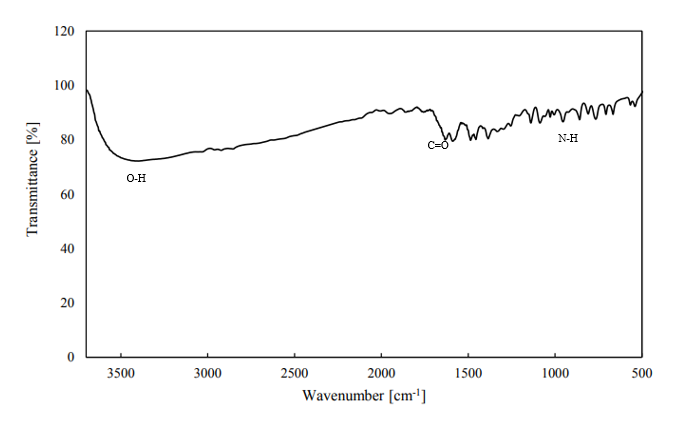


Fig S2. FT-IR spectrum of [Ch][Sal] IL.


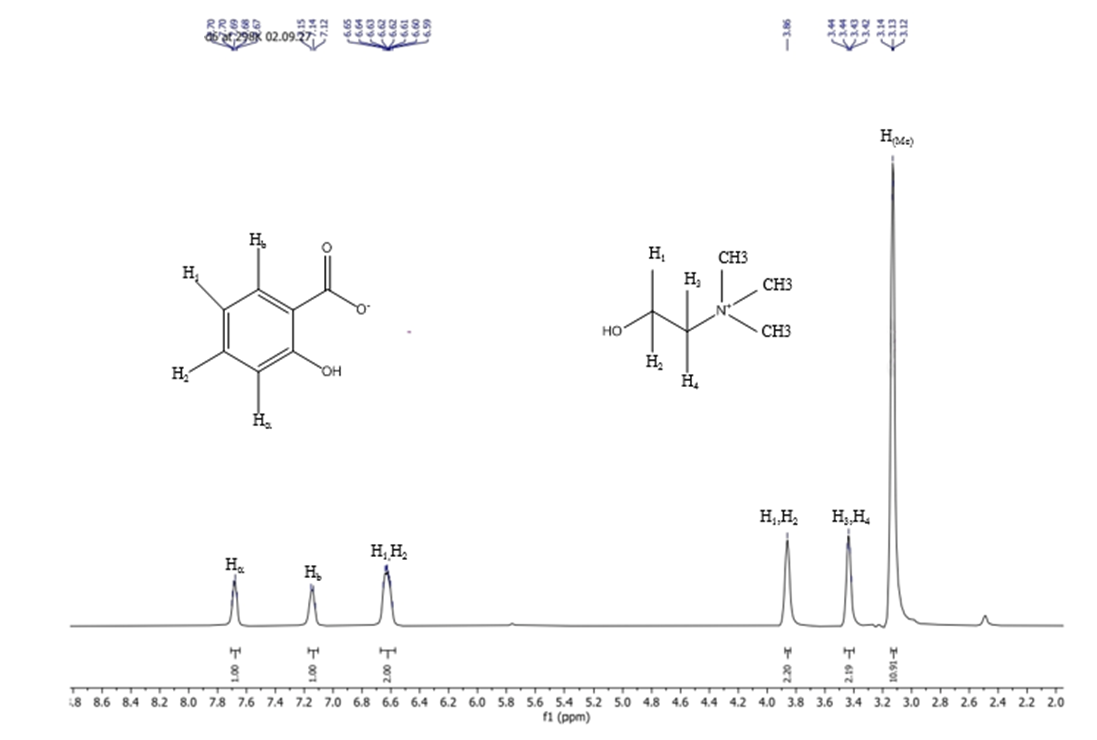


***Fig S3*.** ^1^H-NMR spectrum of the [Ch][Sal] IL.


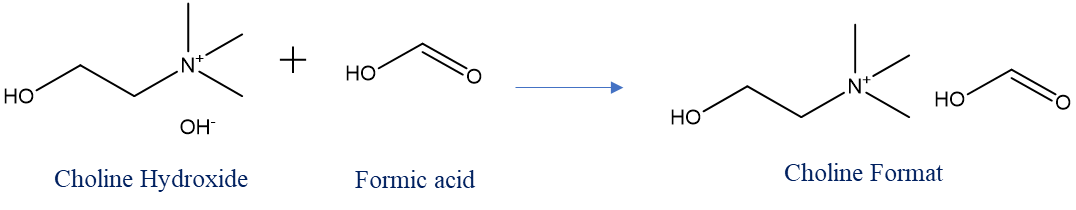


***Fig S4.*** The synthesis plan of the [Ch][For] IL.


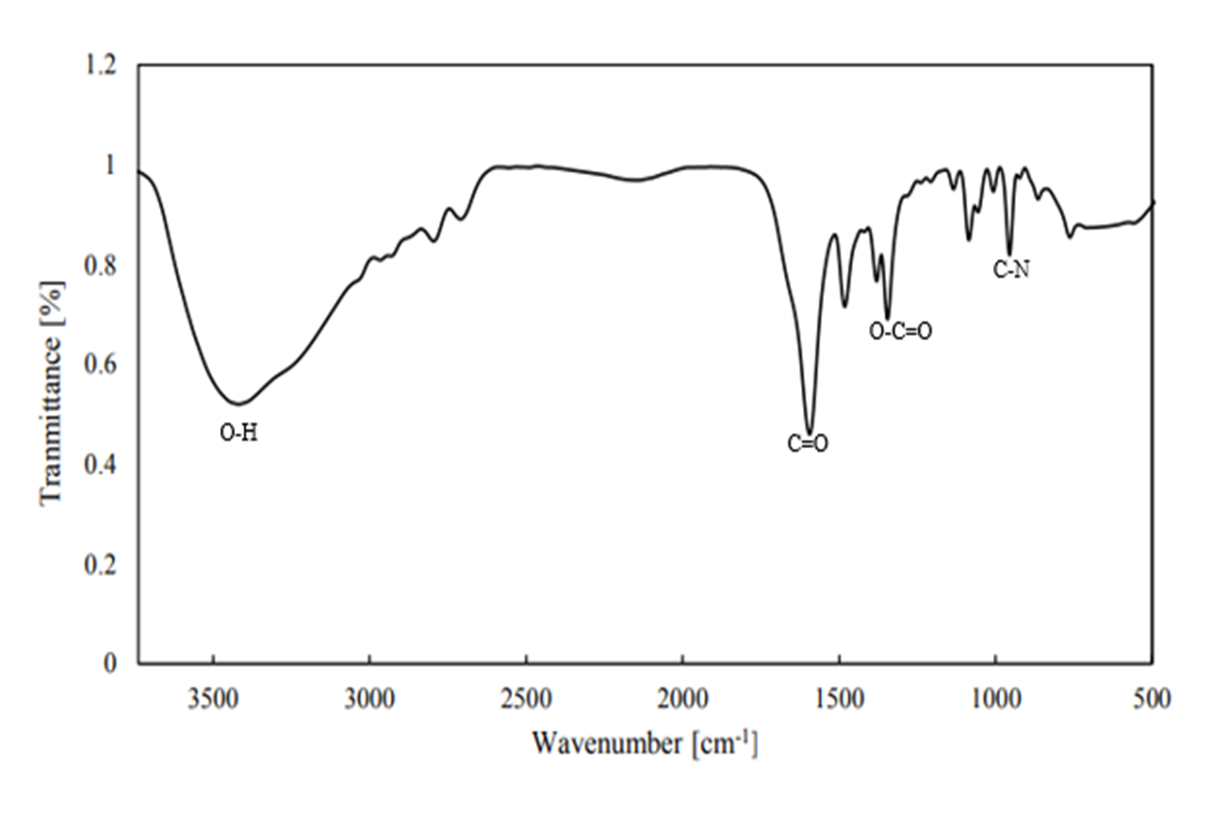


***Figure S5.*** FT-IR spectrum of [Ch][For] IL.


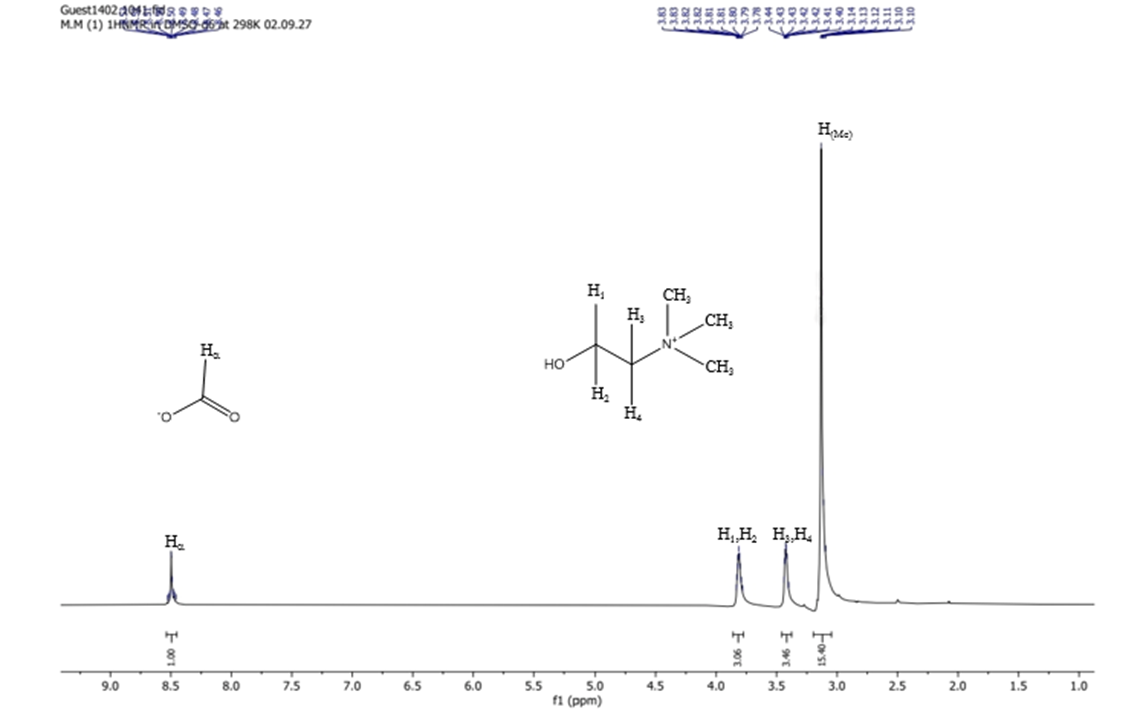


***Figure S6.*** ^1^H-NMR spectrum of [Ch][For] IL.


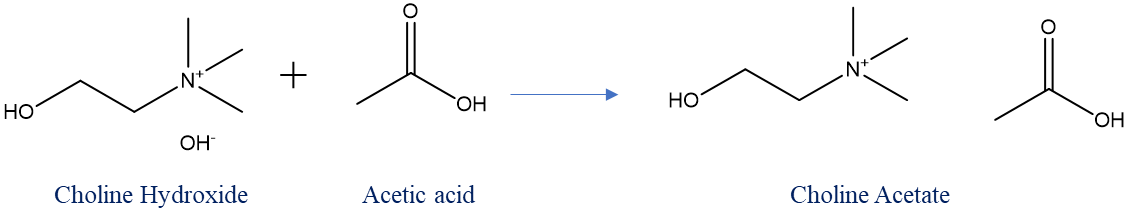


***Figure S7.*** The synthesis plan of the [Ch][Ace] IL.


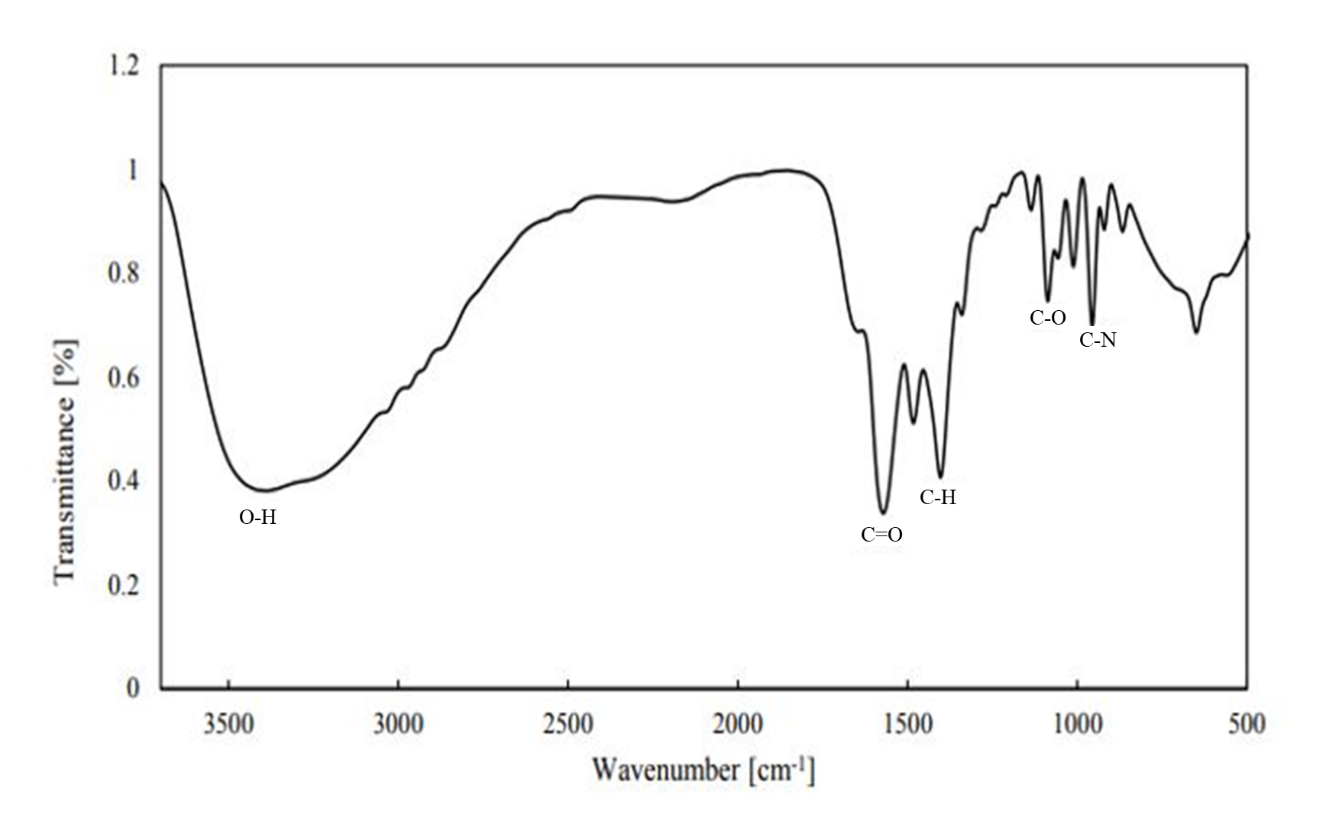


***Figure S8.*** FT-IR spectrum of [Ch][Ace] IL.


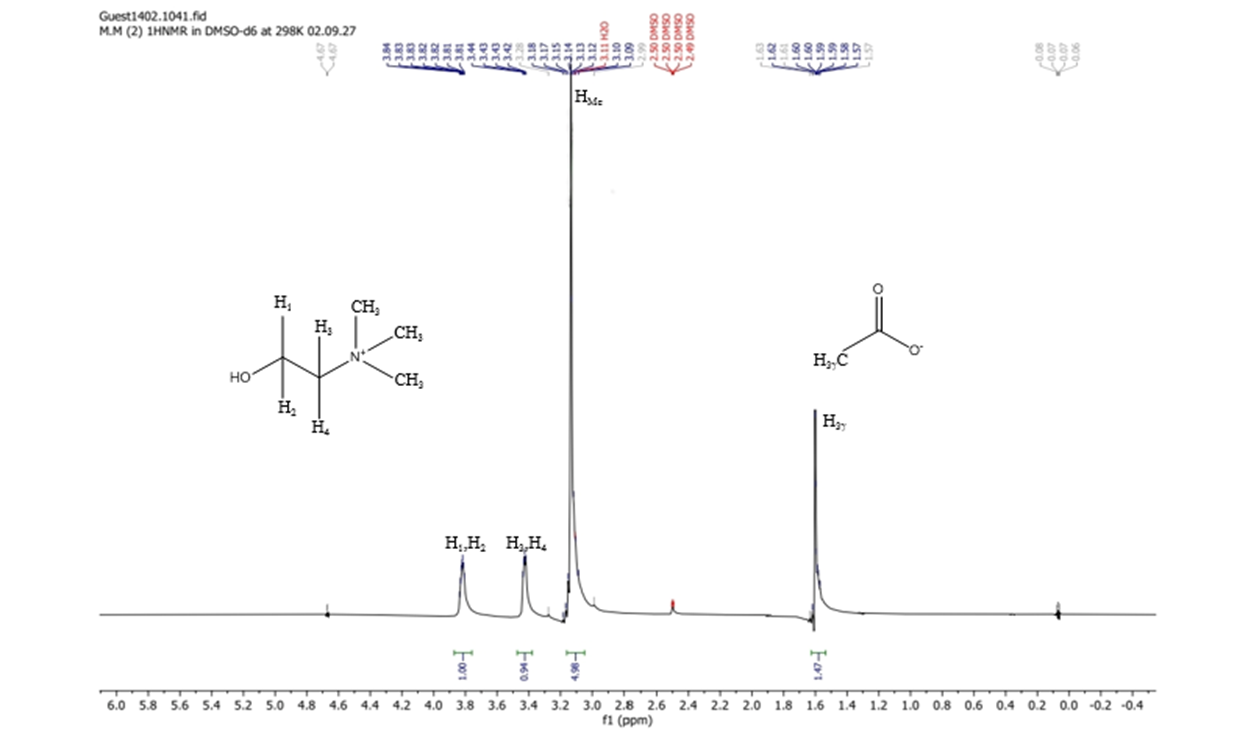


***Figure S9.*** ^1^H-NMR spectrum of [Ch][Ace] IL.

1. *Corresponding author. Tel.: +*98-41-33393094.

   Fax: +98-41-33340191.

   E-mail address: hemayatt@yahoo.com (H. Shekaari). [↑](#footnote-ref-1)
